# Supplementary material for: Educational innovation as a communication strategy in palliative care: A study protocol and preliminary results
Source: PLoS One. 2023 Jun 9;18(6):e0286343. doi: 10.1371/journal.pone.0286343 (PMC10256175; doi:10.1371/journal.pone.0286343)
Supplement: S1 Checklist — The SPIRIT checklist is copyrighted by the SPIRIT Group and is reproduced with permission. (PDF) [file pone.0286343.s001.pdf]

SPIRIT checklist			
Item No.	Section	Description	Checklist
1	Title	Descriptive title identifying the study design, population, interventions, and, if applicable, trial acronym	x
2a	Trial registration	Trial identifier and registry name. If not yet registered, name of intended registry	x
2b		All items from the World Health Organization Trial Registration Data Set	x
3	Protocol version	Date and version identifier	x
4	Funding	Sources and types of financial, material, and other support	x
5a	Roles and responsibilities	Names, affiliations, and roles of protocol contributors	x
5b		Name and contact information for the trial sponsor	x
5c		Role of study sponsor and funders, if any, in study design; collection, management, analysis, and interpretation of data; writing of the report; and the decision to submit the report for publication, including whether they will have ultimate authority over any of these activities	x
5d		Composition, roles, and responsibilities of the coordinating centre, steering committee, endpoint adjudication committee, data management team, and other individuals or groups overseeing the trial, if applicable (see Item 21a for data monitoring committee)	x
6a	Background and rationale	Description of research question and justification for undertaking the trial, including summary of relevant studies (published and unpublished) examining benefits and harms for each intervention	x
6b		Explanation for choice of comparators	
7	Objectives	Specific objectives or hypotheses	x
8	Trial design	Description of trial design including type of trial (eg, parallel group, crossover, factorial, single group), allocation ratio, and framework (eg, superiority, equivalence, noninferiority, exploratory)	x
9	Study setting	Description of study settings (eg, community clinic, academic hospital) and list of countries where data will be collected. Reference to where list of study sites can be obtained	x
10	Eligibility criteria	Inclusion and exclusion criteria for participants. If applicable, eligibility criteria for study centres and individuals who will perform the interventions (eg, surgeons, psychotherapists)	x

11a	Interventions	Interventions for each group with sufficient detail to allow replication, including how and when they will be administered	x
11b		Criteria for discontinuing or modifying allocated interventions for a given trial participant (eg, drug dose change in response to harms, participant request, or improving/worsening disease)	
11c		Strategies to improve adherence to intervention protocols, and any procedures for monitoring adherence (eg, drug tablet return, laboratory tests)	x
11d		Relevant concomitant care and interventions that are permitted or prohibited during the trial	
12	Outcomes	Primary, secondary, and other outcomes, including the specific measurement variable (eg, systolic blood pressure), analysis metric (eg, change from baseline, final value, time to event), method of aggregation (eg, median, proportion), and time point for each outcome. Explanation of the clinical relevance of chosen efficacy and harm outcomes is strongly recommended	
13	Participant timeline	Time schedule of enrolment, interventions (including any run-ins and washouts), assessments, and visits for participants. A schematic diagram is highly recommended (see Figure)	x
14	Sample size	Estimated number of participants needed to achieve study objectives and how it was determined, including clinical and statistical assumptions supporting any sample size calculations	x
15	Recruitment	Strategies for achieving adequate participant enrolment to reach target sample size	
16a	Sequence generation	Method of generating the allocation sequence (eg, computer-generated random numbers), and list of any factors for stratification. To reduce predictability of a random sequence, details of any planned restriction (eg, blocking) should be provided in a separate document that is unavailable to those who enrol participants or assign interventions	
16b	Allocation concealment mechanism	Mechanism of implementing the allocation sequence (eg, central telephone; sequentially numbered, opaque, sealed envelopes), describing any steps to conceal the sequence until interventions are assigned	
16c	Implementation	Who will generate the allocation sequence, who will enrol participants, and who will assign participants to interventions	x

17a	Blinding (masking)	Who will be blinded after assignment to interventions (eg, trial participants, care providers, outcome assessors, data analysts), and how	
17b		If blinded, circumstances under which unblinding is permissible, and procedure for revealing a participant's allocated intervention during the trial	
18a	Data collection methods	Plans for assessment and collection of outcome, baseline, and other trial data, including any related processes to promote data quality (eg, duplicate measurements, training of assessors) and a description of study instruments (eg, questionnaires, laboratory tests) along with their reliability and validity, if known. Reference to where data collection forms can be found, if not in the protocol	x
18b		Plans to promote participant retention and complete follow-up, including list of any outcome data to be collected for participants who discontinue or deviate from intervention protocols	x
19	Data management	Plans for data entry, coding, security, and storage, including any related processes to promote data quality (eg, double data entry; range checks for data values). Reference to where details of data management procedures can be found, if not in the protocol	x
20a	Statistical methods	Statistical methods for analysing primary and secondary outcomes. Reference to where other details of the statistical analysis plan can be found, if not in the protocol	x
20b		Methods for any additional analyses (eg, subgroup and adjusted analyses)	x
20c		Definition of analysis population relating to protocol non-adherence (eg, as randomised analysis), and any statistical methods to handle missing data (eg, multiple imputation)	
21a	Data monitoring	Composition of data monitoring committee (DMC); summary of its role and reporting structure; statement of whether it is independent from the sponsor and competing interests; and reference to where further details about its charter can be found, if not in the protocol. Alternatively, an explanation of why a DMC is not needed	
21b		Description of any interim analyses and stopping guidelines, including who will have access to these interim results and make the final decision to terminate the trial	
22	Harms	Plans for collecting, assessing, reporting, and managing solicited and spontaneously reported adverse events and other unintended effects of trial interventions or trial conduct	
23	Auditing	Frequency and procedures for auditing trial conduct, if any, and whether the process will be independent from investigators and the sponsor	

24	Research ethics approval	Plans for seeking research ethics committee/institutional review board (REC/IRB) approval	x
25	Protocol amendments	Plans for communicating important protocol modifications (eg, changes to eligibility criteria, outcomes, analyses) to relevant parties (eg, investigators, REC/IRBs, trial participants, trial registries, journals, regulators)	x
26a	Consent or assent	Who will obtain informed consent or assent from potential trial participants or authorised surrogates, and how (see Item 32)	x
26b		Additional consent provisions for collection and use of participant data and biological specimens in ancillary studies, if applicable	
27	Confidentiality	How personal information about potential and enrolled participants will be collected, shared, and maintained in order to protect confidentiality before, during, and after the trial	x
28	Declaration of interests	Financial and other competing interests for principal investigators for the overall trial and each study site	x
29	Access to data	Statement of who will have access to the final trial dataset, and disclosure of contractual agreements that limit such access for investigators	x
30	Ancillary and post-trial care	Provisions, if any, for ancillary and post-trial care, and for compensation to those who suffer harm from trial participation	
31a	Dissemination policy	Plans for investigators and sponsor to communicate trial results to participants, healthcare professionals, the public, and other relevant groups (eg, via publication, reporting in results databases, or other data sharing arrangements), including any publication restrictions	x
31b		Authorship eligibility guidelines and any intended use of professional writers	x
31c		Plans, if any, for granting public access to the full protocol, participant-level dataset, and statistical code	
32	Informed consent materials	Model consent form and other related documentation given to participants and authorised surrogates	x
33	Biological specimens	Plans for collection, laboratory evaluation, and storage of biological specimens for genetic or molecular analysis in the current trial and for future use in ancillary studies, if applicable	
