# Supplementary material for: Educational innovation as a communication strategy in palliative care: A study protocol and preliminary results
Source: PLoS One. 2023 Jun 9;18(6):e0286343. doi: 10.1371/journal.pone.0286343 (PMC10256175; doi:10.1371/journal.pone.0286343)
Supplement: S1 Protocol — Approved on February 17, 2022. (PDF) [file pone.0286343.s002.pdf]

Título: "Innovación en la comunicación de cuidados paliativos a través de la experiencia docente"
Proyecto de investigación de la tesis de doctorado de Ana Paula Salas Moreira

1. Definición de objetivos y descripción del proyecto

1.1. Introducción

Los cuidados paliativos ofrecen una respuesta integral para mejorar la calidad de vida de las personas que se enfrentan a enfermedades avanzadas, incurables e incapacitantes (WHO, 2015). Sin embargo, el término "cuidados paliativos" se sigue asociado a los conceptos de "morir", "terminal" y "final de la vida" (Collins, McLachlan, Philip, 2017; Cain, Surbone, Elk, Kagawa-Singer, 2018). Las nuevas generaciones mantienen una visión negativa y alejada de la realidad de lo que son los cuidados paliativos y lo que hacen, y esta forma de conocer genera un discurso que ha sido identificado en varios estudios como una barrera para la implementación de servicios de cuidados paliativos en el sistema nacional de salud (Centeno, Garralda, Carrasco, den Herder-van der Eerden, Aldridge, Stevenson, Meier, & Hasselaar, 2017).

En los últimos años, algunos estudios sociales se han centrado en este problema, tratando de encontrar una forma novedosa de transmitir a la sociedad el mensaje real positivo sobre CP (McIlfatrick et al., 2021; Reigada, Arantzamendi, & Centeno, 2020; Wallerstedt, Benzein, Schildmeijer, & Sandgren, 2019). La comunicación tradicional que se ha desarrollado hasta ahora, a través de medios y comunicación institucional, no parece que esté resultando muy efectiva, dado el estado general de la opinión pública, y resulta necesario, por tanto, encontrar nuevos modos de comunicar.

En un estudio de diagnóstico realizado en España con estudiantes universitarios, se resalta que es positivo contar con los propios estudiantes a la hora de diseñar una estrategia de explicación y difusión del mensaje de cuidados paliativos (CP) (Reigada et al.2021). Por lo tanto, crear un entorno abierto que promueva la discusión del tema entre las personas, a través del arte y la creatividad, fomentando estrategias como la escucha activa y la educación para la sensibilidad intercultural, puede ayudar a desarrollar una conciencia sobre el proceso de vivir con una enfermedad y la conveniencia de contar con los CP (Reigada et al.2021).

El estudio de Reigada et al. (2021) ya apunta a que apostar por la docencia innovadora como estrategia de comunicación pública puede ser la solución adecuada a las necesidades comunicativas actuales. Por otro lado, la educación y la comunicación son dos disciplinas que, más que tener una relación estrecha, tienen una naturaleza inseparable. La educación es un espacio de la comunicación, condicionado por ella, posible a través de ella (Hernández, 1996).

En esta línea, desde el equipo de mensaje de Atlantes Reigada et al. (2021), han estudiado cómo generar conocimiento y sensibilidad sobre los cuidados paliativos entre estudiantes universitarios. A Través de la Investigación-Acción Participativa (IAP), el *Design Thinking* (DT) y adoptando una estrategia de compromiso Público en Investigación e Innovación Responsable (PRRI) han creado una intervención social para promover un aprendizaje transformador, activo y de aprendizaje servicio sobre los cuidados paliativos, con y para los estudiantes de la Universidad de Navarra (UNAV).

Este programa de intervención social se convierte en una asignatura del Instituto CORE Curriculum de la Universidad de Navarra llamada de "Cuidado y Sociedad" y que se iniciará en enero de 2022 con 31 alumnos de que no están en grados de salud porque se entiende que estos son los perfiles que menos sensibilidad tienen

para el tema de los cuidados pero que pueden ser también ellos, los futuros decisores, influenciadores y promotores de un discurso social asertivo, en la sociedad. A través de esta intervención social (la asignatura Cuidado y Sociedad) se busca crear embajadores del mensaje de los CP en el campus universitario UNAV.

El objetivo de este estudio es evaluar la experiencia de aprendizaje de los estudiantes de la asignatura “Cuidado y Sociedad” que busca potenciar las actitudes positivas hacia los cuidados y los cuidados paliativos de los estudiantes universitarios.

1.2. Objetivo
Evaluar la experiencia de aprendizaje de los estudiantes de la asignatura “Cuidado y Sociedad” de la Universidad de Navarra en el año académico 2021/2022.

Objetivos específicos
- Evaluar la aceptabilidad y factibilidad de la asignatura “Cuidado y Sociedad”, como circuito de aprendizaje para disminuir la desinformación y los malentendidos en torno a los cuidados paliativos, fomentado el debate social.
- Explorar los conocimientos y actitudes de los estudiantes universitarios de la asignatura Cuidado y Sociedad.
- Promover a los estudiantes de la UNAV a “embajadores de CP” para que sean los conectores de los valores en torno a los cuidados como es la empatía.

2. Metodología
Estudio de Investigación-Acción, usando múltiples métodos para la recogida de datos (como aplicación de escalas, cuestionarios de satisfacción, inteligencia artificial, grupos de discusión y observación directa). Para el análisis se usarán las transcripciones de los grupos focales, notas de campo, y ejercicios en aula (por ejemplo, los trabajos realizados por los alumnos en cada módulo de la asignatura). Para cada etapa del estudio, presentaremos en detalle los métodos utilizados para la recogida de datos y los respectivos enfoques para el tratamiento de datos (análisis cuantitativo y cualitativo).

2.1 Justificación de los métodos seleccionados
La IAP se define como un método de investigación progresivo de planificación, acción, observación y resultados para comprender y transformar las realidades y prácticas socioeducativas (Lewin, K, 1946). Entendemos que, siendo una metodología participativa, nos ayudará a alcanzar los objetivos propuestos, asumiendo en este estudio una perspectiva constructivista. Es decir, la existencia de múltiples realidades puede resultar de la construcción humana donde, interactivamente, es posible obtener resultados factuales. Aplicando este método de investigación que requiere un análisis constante del proceso, y las interpretaciones intersubjetivas que resultan de ahí, se puede llegar a una realidad válida para ser explicada en un contexto determinado (Denzin & Lincoln, 2013).

La evaluación general de la asignatura se centrará en cuatro etapas:
- Etapa 1 - Evaluación inicial
- Etapa 2 - Evaluación del aprendizaje
- Etapa 3 - Evaluación final
- Etapa 4 - Evaluación a medio plazo

Las cuestiones de investigación serán:

1. ¿Puede la asignatura Cuidado y Sociedad promover el conocimiento de los estudiantes sobre los cuidados y cuidados paliativos?
2. ¿Puede la asignatura Cuidado y Sociedad contribuir para actitudes positivas de los estudiantes hacia los cuidados paliativos?
3. ¿Es posible convertir a los estudiantes de grado en embajadores de cuidados paliativos?

2.2 - Programa de intervención social: Asignatura Cuidado y Sociedad

Esta asignatura se centra en promover el conocimiento, potenciar la empatía y las actitudes positivas de los estudiantes sobre los temas relacionados con el acompañar y cuidar de las personas que sufren por una enfermedad grave y su entorno, de manera interdisciplinar. Es una asignatura de carácter optativo (3 ECTs) del Instituto Core *Curriculum* de la Universidad de Navarra.

En el año académico 2021/2022 se ofertará esta asignatura a un máximo de 30 estudiantes de la Facultad de Ciencias Económicas y Empresariales, Escuela de Arquitectura, Facultad de Educación y Psicología. El objetivo es evaluar y mejorar la asignatura para ser implementada en los próximos años, en UNAV y en otras universidades de España. Por lo tanto, se considera este estudio un estudio piloto, considerando la asignatura Cuidado y Sociedad el prototipo de la intervención social.

Fig. 1 - Resumen de la asignatura Cuidado y Sociedad

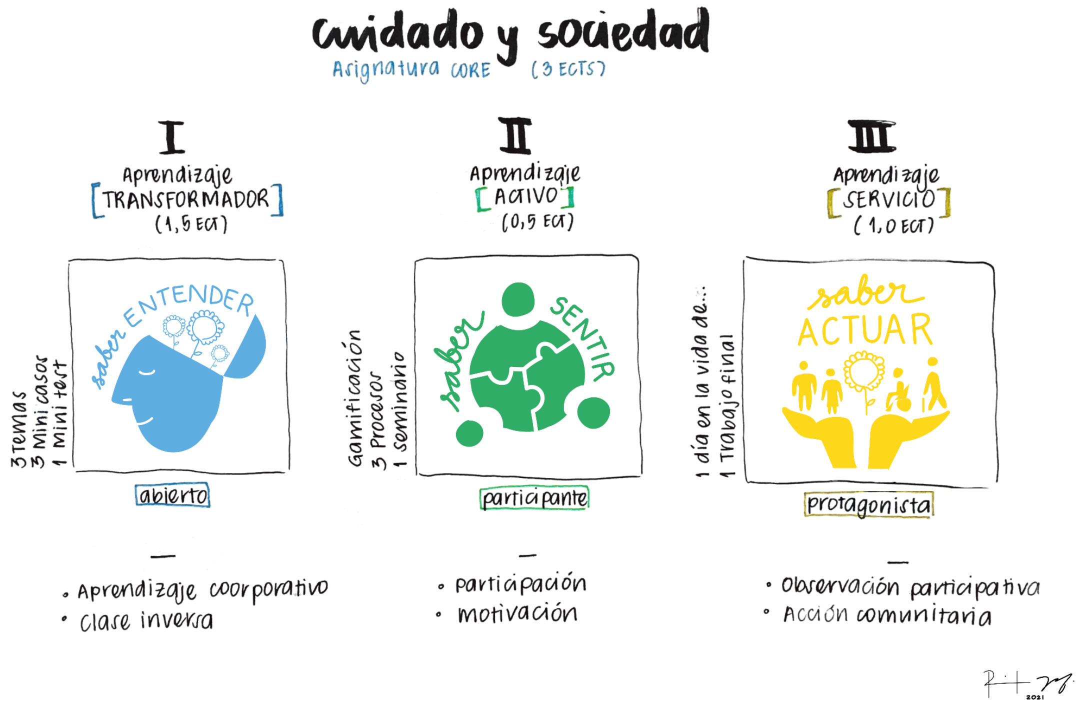

Módulo I - Aprendizaje transformador: abiertos para acompañar y cuidar

Al final del módulo el alumno será capaz de:

- Enumerar las situaciones de enfermedad grave que cursan con intenso sufrimiento;
- Describir cuándo y cómo se acompaña, cuida y alivia y cuando no;
- Estructurar un conjunto de acciones que cooperativamente ayude a cuidar y aliviar a la persona con sufrimiento.

Módulo II - Aprendizaje activo: el alumno participante del cuidar
**Actividad dinámica que consiste en participar en un circuito de autoaprendizaje (juego "Stay Room") donde los alumnos serán protagonistas en construir su propio conocimiento relacionado con el tema del cuidar.*
Al final del módulo el alumno será capaz de:

- Entender cuál y cómo es el proceso de acompañar, cuidar y aliviar el sufrimiento

Módulo III - Aprendizaje servicio: el alumno protagonista del cuidado
**Este módulo promoverá el aprendizaje-servicio. Los alumnos participarán en actividades de acción comunitaria, permitiendo conectar lo que aprenden en módulo I y II con un contexto real y de forma vivencial y reflexivo. Este módulo será realizado contando con la colaboración de TANTAKA y la Unidad de Innovación Social de Navarra.*
Al final del módulo el alumno será capaz de:

- Planificar acciones con base en lo aprendido y con vista a desarrollar sus competencias personales y profesionales;
- Evaluar, establecer y recomendar buenas prácticas en la relación al cuidar de las personas con enfermedad grave.

2.3 - Etapas de la evaluación del programa de intervención social Cuidado y Sociedad
La evaluación de programas de intervención social objetiva medir el grado de adecuación, eficacia y eficiencia del programa, así como, detectar y orientar las mejoras a nuevas soluciones, facilitando el análisis y diseño prospectivo de futuras intervenciones. En este estudio, la evaluación de la intervención se centrará en la factibilidad y aceptabilidad de la asignatura. Se entiende por factibilidad el grado de éxito en cuanto a la implementación y cumplimento de los objetivos propuestos, y la aceptabilidad se refleja a través del *feedback* positivo de los participantes.

Trataremos de realizar la evaluación del programa en general en el tiempo, realizando una evaluación pre-post intervención. Se tendrá en cuenta cuatro momentos de la evaluación: 1) inmediatamente antes del inicio del programa (Evaluación Inicial), 2) durante la experiencia del programa (Evaluación del Aprendizaje) y 3) inmediatamente después de la finalización del programa (Evaluación Final) y 4) 5 meses después de terminar la asignatura Cuidado y Sociedad (Evaluación a medio plazo).

Etapa 1 - Evaluación Inicial (abordaje cuantitativo)

1. Para realizar la evaluación inicial, una semana antes del inicio de la asignatura "Cuidado y Sociedad" (2021/2022), los estudiantes recibirán un correo invitándolos a participar en el estudio. Este correo será enviado a través de la plataforma ADI. Todo los cuestionarios se harán electrónicamente a través del formulario *Google.*
2. Los dos cuestionarios online para rellenar son: un cuestionario que medirá el grado de conocimiento sobre los cuidados paliativos (cuestionario de conocimientos de Cuidados Paliativos) y el otro que mide el nivel de empatía de los estudiantes [Test de Empatía Cognitiva y Afectiva (TECA)]; este último ha sido ya adquirido por ATLANTES para otros estudios.
3. Se asegurará la confidencialidad de los datos y la anonimidad de los estudiantes cuando rellenan el cuestionario. Se pedirá lo siguiente: "Antes de iniciar, por favor indique un código para el cuestionario. Indique el nombre de una ciudad y tres números, por ejemplo, Pamplona123 (NO se puede usar esta ciudad, ni esta combinación de números para su código). Es importante recordar este código, porque debe ser usado en todos los cuestionarios que le enviaremos. Esto nos permitirá comparar respuestas sin identificar personas". Además, se asegurará que el formulario *google* no pida ni muestre el email del remitente.

4. Sin interferir con la evaluación académica del profesor de la asignatura, al final del **módulo I**, a los estudiantes que hayan autorizado el recontacto se les enviarán los enlaces para la segunda ronda del cuestionario de conocimientos sobre cuidados paliativos inicial y el TECA.

Instrumentos: El Cuestionario de Conocimientos de Cuidados Paliativos será desarrollado por el equipo investigador ATLANTES, experto en Cuidados Paliativos (ANEXO B); El Test de Empatía Cognitiva y Afectiva (TECA) es una medida global de empatía con 33 ítem, que presenta una estructura de cuatro factores a evaluar: i) la adopción de perspectivas relativas a la capacidad intelectual o imaginativa de ponerse en el lugar de otra persona, ii) la capacidad de reconocer y reconocer y comprender las emociones, intenciones e impresiones de otras personas, iii) la capacidad de compartir las emociones negativas de otra persona, y iv) la capacidad de compartir las emociones positivas de otra persona.

Participantes:
Estudiantes: Estudiantes inscritos en la asignatura "Cuidado y Sociedad" del Instituto Core Curriculum 2021/2022 de la Universidad de Navarra.

Etapa 2 - Evaluación del Aprendizaje (abordaje cuantitativa y cualitativa)
La asignatura Cuidado y Sociedad se va a realizar en la Universidad de Navarra una vez a la semana, durante dos horas, de enero a abril de 2022. Además de la evaluación pre y post intervención, es importante realizar su evaluación continua. Por lo tanto, en esta etapa, se evaluarán las experiencias de los estudiantes al respecto de los objetivos de cada módulo de la asignatura. Concretamente:

Módulo I Aprendizaje Transformador - Este módulo tiene como objetivo fomentar el autoaprendizaje y el pensamiento crítico de los estudiantes. Son los estudiantes que, mediante trabajo en pequeños grupos y la discusión en gran grupo, transmitirán a los demás conocimientos teórico-prácticos sobre las cuestiones relacionadas con los cuidados paliativos. A través de estudios de casos proporcionados por los docentes, de la discusión general y la clase inversa, al final de este módulo los estudiantes deben conseguir:

1. Describir la situación de enfermedad y cómo esta afecta a las personas.
2. Describir la importancia de acompañar y cuidar.
3. Describir un plan de acción para acompañar y cuidar una persona en situación de enfermedad grave.

Módulo II Aprendizaje Activo - Este módulo ofrece una actividad dinámica, un circuito de autoaprendizaje donde los alumnos serán protagonistas en construir su propio conocimiento relacionado con el tema del cuidar. Se hará un *Stay Room* (similar a un *Escape Room*) que ya ha sido prototipado y testado con los alumnos de la UNAV (aprobación ética 2020.179). Para evaluar la experiencia del *Stay Room* (**Módulo II**), se asignará un momento para el *debriefing* de 10 minutos al final del juego que será grabado por fines de la docencia. Con **consentimiento de los alumnos, las transcripciones de esta grabación serán usadas** para ayudar a analizar los datos.

Se harán tres preguntas a los estudiantes al inicio de la sesión:

1. ¿De 1 a 5, indica el grado de motivación para hacer el juego?
2. ¿De 1 a 5, indica el grado de empatía que consideras tener?
3. Cuéntanos una situación que te genera empatía.

Al final de la sesión, pediremos que los estudiantes respondan a estas preguntas:

4. ¿De 1 a 5, indica si el juego ha cumplido tu expectativa?
5. ¿De 1 a 5, indica el grado de empatía que consideras tener?
6. ¿Qué mensaje te llevas?

Módulo III Aprendizaje servicio - En este módulo se pretende que cada estudiante pase un día con una persona conectada al cuidado (puede ser un profesional de salud, un cuidador, un paciente). Sabiendo que este módulo es evaluado por el docente a través de una **reflexión escrita de los estudiantes**, con el consentimiento previo de los alumnos, este material se analizará para la investigación. Los trabajos de los alumnos deberán ser enviados a los investigadores por los docentes, quienes asegurarán el anonimato de los autores antes de su envío. Las reflexiones pueden realzar los valores de esta experiencia y, por lo tanto, el contenido escrito será analizado por dos investigadores independientes, de forma cualitativa y abierta (análisis temático/codificación abierta).

Participantes:
Estudiantes: Estudiantes inscritos en la asignatura "Cuidado y Sociedad" del Instituto Core Curriculum 2021/2022 de la Universidad de Navarra.

Etapa 3 - Evaluación Final (abordaje cuantitativa y cualitativa)
Los estudiantes recibirán el último set de cuestionarios (conocimientos sobre los cuidados paliativos final y TECA). Utilizando el mismo código de participante seleccionado al inicio del estudio, podrán participar en el estudio rellenando **los cuestionarios** de forma online (formulario *Google*). También se les enviará un enlace para que puedan dar el *feedback* de la asignatura a través a través de un **cuestionario interactivo de satisfacción y compromiso (desarrollado por el grupo de investigación)**, que contempla una pregunta abierta y un compromiso de aprendizaje en relación al cambio de actitud: *"Después de lo que has aprendido en esta asignatura, ¿qué te propones a cambiar en tu actitud en los próximos meses, para mejorar el mensaje de cuidados paliativos?"*. Estos resultados serán codificados con el mismo código de los cuestionarios anteriores.

Las últimas dos clases de la asignatura contemplan dos seminarios donde los estudiantes son preparados para argumentar sobre los cuidados paliativos. Se generará un debate social en aula sobre distintas cuestiones sobre el cuidar, y este debate será grabado por fines de la docencia. El audio de estos discursos será transcrito, garantizando que no aparezca ningún dato que pueda identificar a los alumnos, y será usado para el análisis de la investigación con el consentimiento previo de los estudiantes.

Los docentes, posibles colaboradores, y cinco estudiantes de la asignatura serán invitados a participar en un grupo de discusión para evaluar la experiencia. Se seleccionarán los cinco primeros estudiantes que respondan a este pedido que se hará enviando un email. El objetivo de este grupo discusión es analizar los puntos fuertes y puntos a mejorar del método, contenidos y otros aspectos de la asignatura. Como pequeño incentivo a la participación, se regalará un *voucher* de 20€ Amazon a los estudiantes. Los grupos de discusión serán grabados en formato audio, su contenido será transcrito y analizado por los investigadores de forma confidencial.

Participantes:
Estudiantes: Estudiantes inscritos en la asignatura "Cuidado y Sociedad" del Instituto Core Curriculum 2021/2022 de la Universidad de Navarra.

Docentes: Docentes y colaboradores docentes de la asignatura "Cuidado y Sociedad" del Instituto Core Curriculum, de la Universidad de Navarra.

Etapa 4 - Evaluación a medio plazo
Cinco meses después del término de la asignatura será enviado a los estudiantes, por email, un link con un

cuestionario online para que indiquen en qué medida ha aplicado el compromiso de aprendizaje. Los estudiantes deberán responder a la cuestión *"Te has propuesto cambiar tu actitud para mejorar el mensaje de cuidados paliativos en los últimos meses. ¿Lo has conseguido cumplir? Por favor, escribe un ejemplo"*. En este cuestionario online se utilizará el mismo código de participante seleccionado al inicio del estudio.

3. Análisis

El análisis cuantitativo se realizará con un investigador experto en análisis de datos del grupo ATLANTES. Para dos datos cualitativos se realizará un análisis temático, inductivo, sin códigos predefinidos de las transcripciones audio, notas de observación, y materiales de la asignatura (por ejemplo: trabajos de los estudiantes). Todos los datos se analizarán teniendo en cuenta las cuestiones de investigación, que guiarán la presentación de los resultados, mostrando el fundamento y los aspectos emocionales de la experiencia de los participantes (pensamientos, conocimientos, sentimientos, preconcepciones, expectativas). La fiabilidad de los resultados de la investigación se logrará mediante la triangulación (múltiples métodos, múltiples puntos de vista de los investigadores y múltiples tipos de datos), lo que permitirá explorar diferentes aspectos del problema y contribuir a una interpretación coherente de los datos.

4. Grupo de Investigación

Nombre y rol	Perfil
Ana Paula Salas<br>Doctoranda	Doctoranda en la Facultad de Comunicación, Universidad de Navarra
Carla Reigada<br>Directora	Doctora en Psicología. Investigadora en ATLANTES, Universidad de Navarra.
Beatriz Gómez<br>Directora	Doctora en Comunicación en la Facultad de Comunicación de la Universidad de Navarra. Profesora de comunicación escrita y periodismo literario.

5.Estrategia y plan de comunicación

**Seminario /Jornadas**	Hacer una mañana de jornadas en el ayuntamiento de Pamplona, abierto por zoom, sobre la importancia del cuidado y de los cuidados paliativos. Los ponentes serán miembros del equipo de investigación, y estudiantes de la asignatura Cuidado y Sociedad. Las jornadas se anunciarán a través de los medios de comunicación.	Público General

Para el Público en general

Actividad	Descripción	Público objetivo no experto
**Comunicados de prensa y redes sociales**	La difusión del estudio se realizará a través de las redes sociales, a través de los perfiles del ICS/ATLANTES y Unidad de Innovación Social. El sitio web de la ICS actuará como eje central de toda la información relacionada con el estudio y donde se encontrarán todos los datos de contacto de los miembros del equipo del proyecto.	Público general

	Escribir dos artículos en periódicos reconocidos: Diario de Navarra (periódico o de la Comunidad) y La Razón.<br><br>Realizar un reportaje/pequeño debate con la televisión local (NavarraTV), sobre la importancia del pensamiento crítico en la sociedad, en relación al tema del cuidado y cuidados paliativos.	

Para un público Académico

Actividad	Descripción	Público objetivo experto
**Conferencias**	Enviar un resumen de los resultados (comunicación oral) a la conferencia de investigación de innovación social / Educación	Académicos/investigadores
**Revistas académicas**	Presentar al menos un artículo científico en revistas revisadas por pares (Q1/Q2) como: BMC Palliative Care (IF. 3.815/open Access).	Profesionales sanitarios /académicos /investigadores
**Seminarios de investigación**	Al menos un seminario de investigación que incluya al equipo de investigación y a los estudiantes, que tendrá lugar en la UNAV para presentar y discutir el estudio. Los ponentes serán miembros del equipo de investigación. La conferencia se anunciará a través de los medios de comunicación.	Profesionales sanitarios /académicos /investigadores /estudiantes

6. Consideraciones éticas

El estudio atenderá en todo momento a los principios del Código Europeo de Conducta para la Integridad de la Investigación. El Comité Ético de la UNAV velará por que se respeten los principios éticos de la investigación con seres humanos. Las consideraciones éticas se refieren a: consideraciones éticas específicas para las tres fases del proyecto; y consideraciones generales del estudio en su conjunto.

¡Hola a todos!

Mi nombre es Ana Paula Salas y soy estudiante de la Universidad. Estoy haciendo mi doctorado con el grupo de investigación ATLANTES, y quiero invitarte a formar parte de nuestro estudio "Innovación en la comunicación de cuidados paliativos a través de la experiencia docente".

Lo más importante para mí es **conocer tu experiencia sobre la asignatura "Cuidado y Sociedad"**. Y para hacerlo necesito que los alumnos de la asignatura llenen algunos cuestionarios en **tres momentos** distintos de la asignatura: **antes** de su inicio, **durante** la asignatura, y **después** de finalizadas las clases.

Son cuatro cuestionarios:
1) un **Test de Empatía Cognitiva y Afectiva** (*antes, durante y después*)
2) un cuestionario de **Conocimientos sobre los Cuidados Paliativos inicial** (*antes y durante*)
3) un cuestionario de **Conocimientos sobre los Cuidados Paliativos final** (*después*)
4) un cuestionario de **Satisfacción y compromiso** sobre la asignatura (*después*)

¿Y qué tengo que hacer ahora si decido participar? Es muy sencillo, solamente son **tres pasos** que no te tomarán más de 10 minutos:
Paso 1: Lee la **Hoja de Información** y contáctanos si tienes alguna pregunta.
Paso 2: Rellena estos **DOS cuestionarios** a través de la plataforma Google Forms:
- Conocimientos de cuidados paliativos
- TECA

Paso 3: Para poder enviarte los siguientes pasos del estudio necesito que rellenes esta **autorización de recontacto** a través de este enlace.

Es importante recalcar que **la investigación es independiente a la asignatura.** No interferirá en el desarrollo de las clases, ni repercutirá de ninguna manera en sus notas, ya que es voluntaria. ¡Pero **tu participación es muy valiosa**, y nos sería muy útil para poder mejorar nuestro proyecto!

En la **Hoja de Información** que anexo en este correo podrás encontrar todo lo que necesitas saber sobre nuestro trabajo. No obstante, si tienes alguna duda puedes contactar conmigo (asalas.7@alumni.unav.es) o con Beatriz Gómez (bgomezb@unav.es), quien también es parte del equipo de investigación.

¡Muchas gracias por tu atención! Y recuerda que quedo a tu disposición para cualquier pregunta.

Nos vemos pronto,

Ana Paula Salas

HOJA DE INFORMACIÓN PARA LOS ESTUDIANTES
Cuestionarios

Te invitamos a participar en el estudio de investigación "Innovación en la comunicación de cuidados paliativos a través de la experiencia docente", a los estudiantes de Asignatura "Cuidado Y Sociedad" del Instituto Core Curriculum de la Universidad de Navarra.

Es importante recalcar que, tanto si decides participar como si no lo haces, no habrá repercusión alguna en la nota de la asignatura ya que el docente no sabrá quién participa, ni las respuestas de quiénes lo hagan. Además, todos los datos recogidos en este estudio solamente serán analizados por el equipo investigador cuando finalice la asignatura y las notas estén publicadas.

Códigos
Con el fin de proteger la identidad de los estudiantes que decidan ayudarnos, al inicio de los cuestionarios cada participante creará su propio código. Este código será utilizado en todos los cuestionarios, de forma que el investigador solo recibirá el Google Form con el código (y sin ningún dato que identifique al estudiante). Así garantizamos que en los resultados solamente se usen datos codificados.

Confidencialidad
A todos los que participen se les pedirá confidencialidad de la información. Los estudiantes recibirán los primeros cuestionarios a través del correo electrónico que a su vez serán enviados a través de la plataforma ADI. El correo electrónico de aquellos estudiantes que decidan participar será guardado por los investigadores para así poder enviarles a los siguientes pasos del estudio. Estos correos electrónicos serán guardados aparte, y no estarán ligados de ninguna manera a las respuestas de los cuestionarios ni a sus códigos.

Las respuestas de los cuestionarios serán analizadas solamente cuando la asignatura esté terminada y las notas lanzadas. De tal forma se garantiza que los docentes no recibirán ningún tipo de información relacionada con el estudio. Los consentimientos informados sobre el contacto se destruirán una vez termine el estudio y no estarán asociados de ninguna manera a las respuestas de los cuestionarios. Toda la información recopilada será confidencial en conformidad con la Ley de protección de datos.

Datos personales
Los datos de este estudio se incorporarán a una base de datos o fichero sin datos personales y el acceso estará disponible al personal del estudio. Se mantendrá una copia de seguridad del archivo de datos personales relacionado con los participantes reclutados, en el disco duro del equipamiento informático de la universidad del investigador principal, accesible al investigador principal durante 3 años.

Todos los datos personales serán tratados conforme a las leyes actuales de protección de datos, especialmente al Reglamento (UE) 2016/679 del Parlamento Europeo y del Consejo, de 27 de abril de 2016, relativo a la protección de las personas físicas en lo que respecta al tratamiento de datos personales y a la libre circulación de estos, en adelante RGPD.

V2.2_ESTUDIANTES_08/02/2022

El Responsable del Tratamiento de los datos de la Universidad de Navarra, en cumplimiento del citado RGDP, le informa que si participa en este estudio sus datos personales serán tratados por el equipo investigador exclusivamente para los fines que usted autorice al firmar la hoja de consentimiento. También podrán acceder a los datos autoridades competentes y los miembros del comité ético, si lo considerasen necesario para supervisar la realización del estudio. No será posible identificarle a usted a través de las comunicaciones que pudiera generar este estudio.

Usted es el responsable de la veracidad y corrección de los datos que nos entrega y tiene la facultad de ejercer los derechos de acceso, rectificación, supresión, limitación del tratamiento, portabilidad y de oposición de sus datos de acuerdo con lo dispuesto en la normativa en materia de protección de datos. Para ejercerlos, deberá dirigirse por escrito al delegado de Protección de Datos de la UN a la siguiente dirección postal Campus Universitario, S/N, Edificio Central. 31080 Pamplona (Navarra, España) o a la dirección de correo electrónico dpo@unav.es, adjuntando una fotocopia de su documento nacional de identidad o equivalente. En caso de no estar de acuerdo con el tratamiento de datos realizado o considerar vulnerados sus derechos, tiene derecho a presentar una reclamación ante la Agencia Española de Protección de datos (www.agpd.es).

Tómese su tiempo antes de decidir sobre su participación y consulte con otras personas si lo desea.

Datos de contacto

Somos el grupo de investigación ATLANTES, del Instituto Ciencias y Sociedad de la Universidad de Navarra (UNAV), que busca desarrollar una nueva estrategia para la comunicación de los cuidados paliativos a la sociedad. Le rogamos que lea detenidamente esta información y nos consulte para cualquier duda que pueda tener. Para ello, tendrá que ponerse en contacto con Ana Paula Salas o Beatriz Gómez, en la dirección de correo electrónico asalasmo@unav.es o bgomezb@unav.es.

Agradeciendo de antemano su interés y colaboración.

Atentamente,

Ana Paula Salas

Consentimiento informado para los estudiantes -Recontacto

Estimada/o estudiante de la asignatura Cuidado y Sociedad,

Como ya te contamos, estamos llevando a cabo un estudio titulado “Innovación en la comunicación de cuidados paliativos a través de la experiencia docente”, ¡y para sacarlo adelante necesitamos de tu ayuda!

Para seguir analizando la la experiencia del estudiante que cursa la asignatura "Cuidado y Sociedad (2021/2022)" necesitamos que nos des tu correo electrónico y que nos des la autorización para poder enviarte los siguientes pasos de la investigación a través de este medio.

Es importante recalcar que, tanto si decides participar como si no lo haces, no habrá repercusión alguna e[illegible] la nota de la asignatura, ya que el docente no sabrá quién participa, ni las respuestas de quiénes lo hagan. Además, todos los datos recogidos en este estudio solamente serán analizados por el equipo investigador cuando finalice la asignatura y las notas estén publicadas.

¡Muchas gracias por tu colaboración!

*Obligatorio

1. Nombre y Apellido del alumno/a: *

____

2. Mi email: *

____

Proyecto 2021.204
Apobado CEI Unav: Acta 17.02.2022

3. Autorizo que se guarde mi correo electrónico para que los investigadores puedan recontactar conmigo a través de este medio.

Marca solo un óvalo.

- Sí
- No

4. Fecha *

Ejemplo: 7 de enero del 2019

Este contenido no ha sido creado ni aprobado por Google.

Google Formularios

Conocimientos sobre los Cuidados Paliativos

Estimados alumnos/as:

El objetivo del presente cuestionario es evaluar su conocimiento sobre cuidados paliativos. Le agradecemos que exprese con sinceridad sus criterios, pues esta información es completamente confidencial. Para efectos de la evaluación de la asignatura Cuidado y Sociedad, y para efectos de la investigación, le pedimos que rellene el cuestionario antes del curso, después del Módulo I de la asignatura y cuando termine del curso. No le tomará más de 10 minutos.

Este cuestionario forma parte del proyecto "Innovación en la comunicación de cuidados paliativos a través de la experiencia docente", que busca valorar si esta asignatura es una forma eficiente de enviar el mensaje de los cuidados paliativos a la sociedad. Ana Paula Salas es la investigadora principal, y puedes contactar con ella con este correo: asalas.7@alumni.unav.es.

*Obligatorio

1. Antes de iniciar, por favor indique un código para el cuestionario. Indique el nombre de una ciudad y tres números, por ejemplo, Pamplona123 (NO se puede usar esta ciudad ni esta combinación de números para su código). Es importante recordar este código, porque debe s[illegible] usado en todos los cuestionarios que le enviaremos. Esto nos permitirá comparar respuestas [illegible] identificar personas. *

____

Cuestionario

Le agradecemos que exprese con sinceridad sus criterios, pues esta información es completamente confidencial.

Proyecto 2021.204
Apobado CEI Unav: Acta 17.02.2022

2. Seleccione los aspectos que caracterizan los Cuidados Paliativos:

Selecciona todos los que correspondan.

- [ ] Dirigidos a disminuir el sufrimiento y elevar la calidad de vida
- [ ] No se deben ofrecer en el hogar
- [ ] Ofrecer apoyo a los pacientes a llevar una vida lo más activa
- [ ] La familia debe participar siempre en los cuidados paliativos
- [ ] Los aspectos no espirituales no son parte de los cuidados paliativos
- [ ] Se ofrecen para retardar la muerte de los pacientes terminales

3. Seleccione las respuestas que le parezcan correctas: "Los cuidados paliativos incluyen a los pacientes..."

Selecciona todos los que correspondan.

- [ ] Con enfermedades no oncológicas crónicas en estadios avanzados
- [ ] Con demencia en estado avanzado
- [ ] Con VIH/SIDA
- [ ] Con Insuficiencia Renal Crónica Terminal
- [ ] Con Enfermedad Pulmonar Obstructiva Crónica (EPOC) o con otra insuficiencia respiratoria
- [ ] Con enfermedades oncológicas en estado terminal
- [ ] Con pronóstico de vida limitado (inferior a 6 meses)

4. Seleccione las respuestas que le parezcan verdaderas: "En los cuidados paliativos se debe ten en cuenta..."

Selecciona todos los que correspondan.

- [ ] Visión integral e individualizada de los pacientes
- [ ] Solo los brindan los médicos de la atención primaria
- [ ] Un médico con conocimientos puede afrontar solo el proceso paliativo
- [ ] Intervenir tanto sobre el paciente como sobre la familia
- [ ] Debe ser atendido por diferentes especialistas según la necesidad
- [ ] Carácter interdisciplinario
- [ ] Pueden realizarse sin preparación previa del equipo de salud
- [ ] Se necesita mucho más que deseos de hacer el bien
- [ ] Intervención en todos los niveles de atención asistenciales
- [ ] Solo importa la voluntad del paciente y no de los cuidadores

5. De las siguientes situaciones seleccione aquellas que sean susceptibles a recibir cuidados paliativos:

Selecciona todos los que correspondan.

- [ ] Varón de 54 años con cáncer de pulmón en fase avanzada, sin expectativas de curación
- [ ] Mujer de 89 años con Insuficiencia Cardiaca avanzada que no mejora pese a tener el tratamiento adecuado
- [ ] Niño de 4 años con enfermedad de generativa en fase avanzada sin tratamiento curativo conocido
- [ ] Ninguno de los anteriores es susceptible de recibir cuidados paliativos

6. Defina en una frase qué son los cuidados paliativos:

____

____

____

____

____

7. Fecha:

Ejemplo: 7 de enero del 2019

¡Muchas gracias!

Este contenido no ha sido creado ni aprobado por Google.

Google Formularios

Proyecto 2021.204
Apobado CEI Unav: Acta 17.02.2022

Conocimientos sobre los Cuidados Paliativos (Final)

Estimados alumnos/as:

El objetivo del presente cuestionario es evaluar su conocimiento sobre cuidados paliativos. Le agradecemos que exprese con sinceridad sus criterios, pues esta información es completamente confidencial. Para efectos de la evaluación de la asignatura Cuidado y Sociedad, y para efectos de la investigación, le pedimos que rellene el cuestionario antes del curso, después del Módulo I de la asignatura y cuando termine del curso. No le tomará más de 10 minutos.

Este cuestionario forma parte del proyecto "Innovación en la comunicación de cuidados paliativos a través de la experiencia docente", que busca valorar si esta asignatura es una forma eficiente de enviar el mensaje de los cuidados paliativos a la sociedad. Ana Paula Salas es la investigadora principal, y puedes contactar con ella con este correo: asalas.7@alumni.unav.es.

*Obligatorio

1. Por favor escriba el código de su cuestionario (el mismo que creó en el cuestionario inicial): *

____

Cuestionario

Le agradecemos que exprese con sinceridad sus criterios, pues esta información es completamente confidencial.

2. Seleccione los aspectos que caracterizan los Cuidados Paliativos:

Selecciona todos los que correspondan.

- [ ] Dirigidos a disminuir el sufrimiento y elevar la calidad de vida
- [ ] No se deben ofrecer en el hogar
- [ ] Ofrecer apoyo a los pacientes a llevar una vida lo más activa
- [ ] La familia debe participar siempre en los cuidados paliativos
- [ ] Los aspectos no espirituales no son parte de los cuidados paliativos
- [ ] Se ofrecen para retardar la muerte de los pacientes terminales

Proyecto 2021.204
Apobado CEI Unav: Acta 17.02.2022

3. Seleccione las respuestas que le parezcan correctas: "Los cuidados paliativos incluyen a los pacientes..."

Selecciona todos los que correspondan.

- [ ] Con enfermedades no oncológicas crónicas en estadios avanzados
- [ ] Con demencia en estado avanzado
- [ ] Con VIH/SIDA
- [ ] Con Insuficiencia Renal Crónica Terminal
- [ ] Con Enfermedad Pulmonar Obstructiva Crónica (EPOC) o con otra insuficiencia respiratoria
- [ ] Con enfermedades oncológicas en estado terminal
- [ ] Con pronóstico de vida limitado (inferior a 6 meses)

4. Seleccione las respuestas que le parezcan verdaderas: "En los cuidados paliativos se debe ten en cuenta..."

Selecciona todos los que correspondan.

- [ ] Visión integral e individualizada de los pacientes
- [ ] Solo los brindan los médicos de la atención primaria
- [ ] Un médico con conocimientos puede afrontar solo el proceso paliativo
- [ ] Intervenir tanto sobre el paciente como sobre la familia
- [ ] Debe ser atendido por diferentes especialistas según la necesidad
- [ ] Carácter interdisciplinario
- [ ] Pueden realizarse sin preparación previa del equipo de salud
- [ ] Se necesita mucho más que deseos de hacer el bien
- [ ] Intervención en todos los niveles de atención asistenciales
- [ ] Solo importa la voluntad del paciente y no de los cuidadores

5. De las siguientes situaciones seleccione aquellas que sean susceptibles a recibir cuidados paliativos:

Selecciona todos los que correspondan.

- [ ] Varón de 54 años con cáncer de pulmón en fase avanzada, sin expectativas de curación
- [ ] Mujer de 89 años con Insuficiencia Cardiaca avanzada que no mejora pese a tener el tratamiento adecuado
- [ ] Niño de 4 años con enfermedad de generativa en fase avanzada sin tratamiento curativo conocido
- [ ] Ninguno de los anteriores es susceptible de recibir cuidados paliativos

6. Califique la necesidad de formación de futuros profesionales no sanitarios en el área de los cuidados paliativos:

Selecciona todos los que correspondan.

- [ ] Innecesaria
- [ ] Poco necesaria
- [ ] Necesaria
- [ ] Muy necesaria
- [ ] No tengo opinión al respecto

7. ¿Cuál ha sido su experiencia en esta asignatura?

Proyecto 2021.204
Apobado CEI Unav: Acta 17.02.2022

8. ¿Cuál es el mensaje que se lleva de los cuidados paliativos?

9. Fecha:

Ejemplo: 7 de enero del 2019

¡Muchas gracias!

Este contenido no ha sido creado ni aprobado por Google.

Google Formularios

TECA (inicio) Se han guardado todos los cambios en Drive Enviar

Preguntas Respuestas Configuración

Escala de la Empatía TECA

Las siguientes frases se refieren a sus sentimientos y pensamientos en distintas situaciones. Indique cómo le describe cada una, eligiendo la puntuación de 1 a 5. Lea cada frase cuidadosamente antes de responder. Conteste honestamente con lo que más se identifique (no hay respuestas correctas o incorrectas). No deje ninguna frase sin contestar.

Para efectos de la evaluación de la asignatura Cuidado y Sociedad, y para efectos de la investigación, le pedimos que rellene el cuestionario antes del curso, después del Módulo I de la asignatura y cuando termine del curso. No le tomará más de 10 minutos.

Este cuestionario forma parte del proyecto “Innovación en la comunicación de cuidados paliativos a través de la experiencia docente”, que busca valorar si esta asignatura es una forma eficiente de enviar el mensaje de los cuidados paliativos a la sociedad. Ana Paula Salas es la investigadora principal, y puedes contactar con ella con este correo: asalas.7@alumni.unav.es.

Código del participante. Usa el mismo que usaste en el Cuestionario de Conocimientos (nombre de una ciudad y tres números). *

Texto de respuesta corta

1.- Me resulta fácil darme cuenta de las intenciones de los que me rodean. *

- 1. Totalmente en desacuerdo
- 2. Algo en desacuerdo
- 3. Neutro
- 4. Algo de acuerdo
- 5. Totalmente de acuerdo

2.- Me siento bien si los demás se divierten. *

- 1. Totalmente en desacuerdo
- 2. Algo en desacuerdo
- 3. Neutro
- 4. Algo de acuerdo
- 5. Totalmente de acuerdo

3.- No me pongo triste sólo porque un amigo o amiga lo esté. *

- 1. Totalmente en desacuerdo
- 2. Algo en desacuerdo
- 3. Neutro
- 4. Algo de acuerdo
- 5. Totalmente de acuerdo

Proyecto 2021.204
Apobado CEI Unav: Acta 17.02.2022

4.- Si un amigo o amiga consigue un trabajo muy deseado, me entusiasmo con él o ella. *

1. Totalmente en desacuerdo
2. Algo en desacuerdo
3. Neutro
4. Algo de acuerdo
5. Totalmente de acuerdo

5.- Me afecta demasiado ver programas de televisión donde se muestran los problemas de otras personas. *

1. Totalmente en desacuerdo
2. Algo en desacuerdo
3. Neutro
4. Algo de acuerdo
5. Totalmente de acuerdo

Proyecto 2021.204
Apobado CEI Unav: Acta 17.02.2022

6.- Antes de tomar una decisión intento tener en cuenta todos los puntos de vista. *

- 1. Totalmente en desacuerdo
- 2. Algo en desacuerdo
- 3. Neutro
- 4. Algo de acuerdo
- 5. Totalmente de acuerdo

7.- Rara vez reconozco cómo se siente una persona con sólo mirarla. *

- 1. Totalmente en desacuerdo
- 2. Algo en desacuerdo
- 3. Neutro
- 4. Algo de acuerdo
- 5. Totalmente de acuerdo

8.- Me afecta poco escuchar desgracias sobre personas desconocidas. *

- 1. Totalmente en desacuerdo
- 2. Algo en desacuerdo
- 3. Neutro
- 4. Algo de acuerdo
- 5. Totalmente de acuerdo

9.- Me alegra ver que alguien nuevo se encuentra a gusto en nuestro grupo. *

- 1. Totalmente en desacuerdo
- 2. Algo en desacuerdo
- 3. Neutro
- 4. Algo de acuerdo
- 5. Totalmente de acuerdo

10.- Me es difícil entender cómo se siente una persona ante una situación que no he vivido. *

- 1. Totalmente en desacuerdo
- 2. Algo en desacuerdo
- 3. Neutro
- 4. Algo de acuerdo
- 5. Totalmente de acuerdo

11.- Cuando alguien cercano se ha portado mal conmigo intento entender sus motivos. *

- 1. Totalmente en desacuerdo
- 2. Algo en desacuerdo
- 3. Neutro
- 4. Algo de acuerdo
- 5. Totalmente de acuerdo

12.- Menos que se trate de algo muy grave, me cuesta llorar con lo que les sucede a otros. *

- 1. Totalmente en desacuerdo
- 2. Algo en desacuerdo
- 3. Neutro
- 4. Algo de acuerdo
- 5. Totalmente de acuerdo

13.- Reconozco fácilmente cuando alguien está de mal humor. *

- 1. Totalmente en desacuerdo
- 2. Algo en desacuerdo
- 3. Neutro
- 4. Algo de acuerdo
- 5. Totalmente de acuerdo

14.- No siempre me doy cuenta cuando la persona que tengo al lado se siente mal. *

- 1. Totalmente en desacuerdo
- 2. Algo en desacuerdo
- 3. Neutro
- 4. Algo de acuerdo
- 5. Totalmente de acuerdo

15.- Intento ponerme en el lugar de los demás para saber cómo actuarán. *

- 1. Totalmente en desacuerdo
- 2. Algo en desacuerdo
- 3. Neutro
- 4. Algo de acuerdo
- 5. Totalmente de acuerdo

Proyecto 2021.204
Apobado CEI Unav: Acta 17.02.2022

16.- Cuando a alguien le sucede algo bueno, siento alegría. *

1. Totalmente en desacuerdo
2. Algo en desacuerdo
3. Neutro
4. Algo de acuerdo
5. Totalmente de acuerdo

17.- Cuando tengo una opinión formada, no presto mucha atención a los argumentos de los demás. *

1. Totalmente en desacuerdo
2. Algo en desacuerdo
3. Neutro
4. Algo de acuerdo
5. Totalmente de acuerdo

18.- A veces sufro más con las desgracias de otras personas que ellas mismas. *

- 1. Totalmente en desacuerdo
- 2. Algo en desacuerdo
- 3. Neutro
- 4. Algo de acuerdo
- 5. Totalmente de acuerdo

19.- Me siento feliz sólo con ver felices a otras personas. *

- 1. Totalmente en desacuerdo
- 2. Algo en desacuerdo
- 3. Neutro
- 4. Algo de acuerdo
- 5. Totalmente de acuerdo

20.- Cuando alguien tiene un problema intento imaginarme cómo me sentiría si estuviera en su situación. *

- 1. Totalmente en desacuerdo
- 2. Algo en desacuerdo
- 3. Neutro
- 4. Algo de acuerdo
- 5. Totalmente de acuerdo

21.- No siento especial alegría si alguien me cuenta que ha tenido un golpe de suerte. *

- 1. Totalmente en desacuerdo
- 2. Algo en desacuerdo
- 3. Neutro
- 4. Algo de acuerdo
- 5. Totalmente de acuerdo

Proyecto 2021.204
Apobado CEI Unav: Acta 17.02.2022

22.- Cuando veo que alguien recibe un regalo, no puedo reprimir una sonrisa. *

- 1. Totalmente en desacuerdo
- 2. Algo en desacuerdo
- 3. Neutro
- 4. Algo de acuerdo
- 5. Totalmente de acuerdo

23.- No puedo evitar llorar con los testimonios de personas desconocidas. *

- 1. Totalmente en desacuerdo
- 2. Algo en desacuerdo
- 3. Neutro
- 4. Algo de acuerdo
- 5. Totalmente de acuerdo

24.- Cuando conozco gente nueva me doy cuenta de la impresión que se han llevado de mi. *

- 1. Totalmente en desacuerdo
- 2. Algo en desacuerdo
- 3. Neutro
- 4. Algo de acuerdo
- 5. Totalmente de acuerdo

25.- Cuando mis amigos o amigas me cuentan que les va bien, no le doy mucha importancia. *

- 1. Totalmente en desacuerdo
- 2. Algo en desacuerdo
- 3. Neutro
- 4. Algo de acuerdo
- 5. Totalmente de acuerdo

Proyecto 2021.204
Apobado CEI Unav: Acta 17.02.2022

26.- Encuentro difícil ver las cosas desde el punto de vista de otras personas. *

- 1. Totalmente en desacuerdo
- 2. Algo en desacuerdo
- 3. Neutro
- 4. Algo de acuerdo
- 5. Totalmente de acuerdo

27.- Entender cómo se siente otra persona es algo muy fácil para mí. *

- 1. Totalmente en desacuerdo
- 2. Algo en desacuerdo
- 3. Neutro
- 4. Algo de acuerdo
- 5. Totalmente de acuerdo

Proyecto 2021.204
Apobado CEI Unav: Acta 17.02.2022

28.- No soy de esas personas que se deprimen con los problemas de los demás. *

1. Totalmente en desacuerdo
2. Algo en desacuerdo
3. Neutro
4. Algo de acuerdo
5. Totalmente de acuerdo

29.- Intento comprender mejor a mis amigos y amigas mirando las situaciones desde su perspectiva. *

1. Totalmente en desacuerdo
2. Algo en desacuerdo
3. Neutro
4. Algo de acuerdo
5. Totalmente de acuerdo

30.- Me considero una persona fría porque no me conmuevo fácilmente. *

- 1. Totalmente en desacuerdo
- 2. Algo en desacuerdo
- 3. Neutro
- 4. Algo de acuerdo
- 5. Totalmente de acuerdo

31.- Me doy cuenta cuando las personas cercanas a mí están especialmente contentas sin que me hayan contado el motivo. *

- 1. Totalmente en desacuerdo
- 2. Algo en desacuerdo
- 3. Neutro
- 4. Algo de acuerdo
- 5. Totalmente de acuerdo

Proyecto 2021.204
Apobado CEI Unav: Acta 17.02.2022

32.- Me resulta difícil ponerme en el lugar de personas con las que no estoy de acuerdo. *

- 1. Totalmente en desacuerdo
- 2. Algo en desacuerdo
- 3. Neutro
- 4. Algo de acuerdo
- 5. Totalmente de acuerdo

33.- Me doy cuenta cuando alguien intenta esconder sus verdaderos sentimientos. *

- 1. Totalmente en desacuerdo
- 2. Algo en desacuerdo
- 3. Neutro
- 4. Algo de acuerdo
- 5. Totalmente de acuerdo

Valoración de la asignatura Cuidado y Sociedad

Por favor, valora la experiencia y déjanos alguna recomendación para mejorar. Muchas gracias por tu tiempo.

1. Código de participante (el mismo usado en los cuestionarios: ciudad + 3 números)

____

2. Valora la experiencia de esta asignatura:

Marca solo un óvalo.

	1	2	3	4	5	
Muy mala	◯	◯	◯	◯	◯	Muy buena

3. Valora la metodología de enseñanza:

Marca solo un óvalo.

	1	2	3	4	5	
Muy mala	◯	◯	◯	◯	◯	Muy buena

Proyecto 2021.204
Apobado CEI Unav: Acta 17.02.2022

4. Menciona una de las cosas más importantes que hayas aprendido:

5. Cómo crees que se podría mejorar la asignatura:

6. Después lo que has aprendido, ¿qué te propones cambiar en tu actitud en los próximos meses para mejorar el mensaje de los cuidados paliativos?

Este contenido no ha sido creado ni aprobado por Google.

Google Formularios

HOJA DE INFORMACIÓN PARA LOS ESTUDIANTES
Material de Clase

Te invitamos a participar en el estudio de investigación “Innovación en la comunicación de cuidados paliativos a través de la experiencia docente”, a los estudiantes de asignatura “Cuidado y Sociedad” del Instituto Core Curriculum de la Universidad de Navarra.

Es importante recalcar que, tanto si decides participar como si no lo haces, no habrá repercusión alguna en la asignatura. El docente no sabrá quién participa, ni las respuestas de quiénes lo hagan. Además, todos los datos recogidos en este estudio solamente serán analizados por el equipo investigador cuando finalice la asignatura y las notas estén publicadas.

¿En qué consiste su colaboración?
Queremos que **nos compartas tu experiencia sobre la asignatura “Cuidado y Sociedad (2021/2022)”**. Para ello, nos gustaría que nos dieras tu consentimiento para acceder a los siguientes materiales de clase: los trabajos escritos y las transcripciones de audio.

1) **Trabajos escritos:** Tu colaboración consiste en **permitir que tus trabajos escritos en clase sean utilizados para la investigación**, asegurando que tu nombre no esté asociado de ninguna manera. Se le pedirá a los docentes que nos entreguen el material sin ningún dato que identifique a los alumnos.

2) **Transcripciones de audio:** Durante la asignatura, ciertas actividades (debriefing posterior a la dinámica del “Stay Room” y el discurso social creado en clase para el módulo III) fueron grabadas con fines de la docencia. Estas grabaciones han sido transcritas por un docente asegurando que no contienen ningún dato personal de los alumnos. Tu colaboración consiste en **permitir que tengamos acceso a esas transcripciones**.

Confidencialidad
A todos los que participen se les pedirá confidencialidad de la información. En todo momento se respetará la confidencialidad de sus datos. Estos materiales no estarán asociadas al nombre de los alumnos, ni estarán enlazados de ninguna manera a los códigos de los cuestionarios. Por lo tanto, los resultados serán siempre publicados garantizando el anonimato.

Este consentimiento informado se recogerá el último día de clases, y permanecerá en un sobre cerrado hasta que la asignatura finalice y las notas sean publicadas. Los consentimientos informados no se guardarán. Se destruirán una vez termine la asignatura. Toda la información recopilada será confidencial en conformidad con la Ley de protección de datos.

Datos personales
Los datos de este estudio se incorporarán a una base de datos o fichero sin datos personales y el acceso estará disponible al personal del estudio. Se mantendrá una copia de seguridad del archivo de datos personales relacionado con los participantes reclutados, en el disco duro del equipamiento informático de la universidad del investigador principal, accesible al investigador principal durante 3 años.

Todos los datos personales serán tratados conforme a las leyes actuales de protección de datos, especialmente al Reglamento (UE) 2016/679 del Parlamento Europeo y del Consejo, de 27 de abril de 2016, relativo a la protección de las personas físicas en lo que respecta al tratamiento de datos personales y a la libre circulación de estos, en adelante RGPD.

El Responsable del Tratamiento de los datos de la Universidad de Navarra, en cumplimiento del

citado RGDP, le informa que si participa en este estudio sus datos personales serán tratados por el equipo investigador exclusivamente para los fines que usted autorice al firmar la hoja de consentimiento. También podrán acceder a los datos autoridades competentes y los miembros del comité ético, si lo considerasen necesario para supervisar la realización del estudio. No será posible identificarle a usted a través de las comunicaciones que pudiera generar este estudio.

Usted es el responsable de la veracidad y corrección de los datos que nos entrega y tiene la facultad de ejercer los derechos de acceso, rectificación, supresión, limitación del tratamiento, portabilidad y de oposición de sus datos de acuerdo con lo dispuesto en la normativa en materia de protección de datos. Para ejercerlos, deberá dirigirse por escrito al delegado de Protección de Datos de la UN a la siguiente dirección postal Campus Universitario, S/N, Edificio Central. 31080 Pamplona (Navarra, España) o a la dirección de correo electrónico dpo@unav.es, adjuntando una fotocopia de su documento nacional de identidad o equivalente. En caso de no estar de acuerdo con el tratamiento de datos realizado o considerar vulnerados sus derechos, tiene derecho a presentar una reclamación ante la Agencia Española de Protección de datos (www.agpd.es).

Tómese su tiempo antes de decidir sobre su participación y consulte con otras personas si lo desea. Si acepta participar en este grupo de diseño, se le pedirá que rellene unos datos personales como edad, género, nacionalidad, grado que cursa y su firma en el consentimiento informado adjunto a esta información.

Datos de contacto

Somos el grupo de investigación ATLANTES, del Instituto Ciencias y Sociedad de la Universidad de Navarra (UNAV), que busca desarrollar una nueva estrategia para la comunicación de los cuidados paliativos a la sociedad. Le rogamos que lea detenidamente esta información y nos consulte para cualquier duda que pueda tener antes de firmar el consentimiento informado. Para ello, tendrá que ponerse en contacto con Ana Paula Salas o Beatriz Gómez, en la dirección de correo electrónico asalasmo@unav.es o bgomezb@unav.es.

Agradeciendo de antemano su interés y colaboración.

Atentamente,

Ana Paula Salas

CONSENTIMIENTO INFORMADO PARA LOS ESTUDIANTES
Material de clase

Yo (por favor escriba su nombre),

____, he recibido información clara sobre el estudio que lleva por título **Innovación en la comunicación de cuidados paliativos a través de la experiencia docente** y acepto participar. Entiendo que los datos aportados serán tratados de forma confidencial, salvaguardando mi identidad en los informes o publicaciones que se puedan generar con los resultados del estudio. Entiendo también que me es solicitada la confidencialidad de la información que se genere en la asignatura.

Por favor seleccione una opción:

Autorizo que se utilicen los trabajos escritos producidos en clase, garantizando que no estarán asociados de ninguna manera a datos que puedan identificar al estudiante.

SI ___ No ___

Autorizo que se utilicen las transcripciones de las grabaciones de clase, garantizando que se eliminará cualquier información que identifique a estudiantes.

SI ___ No ___

Declaro que he tenido el tiempo y la oportunidad para realizar preguntas y plantear las dudas que poseyera, y que todas las preguntas fueron respondidas a mi entera satisfacción.

Entiendo que mi participación es libre y voluntaria y que, si decido no participar más en el estudio en algún momento, no tendré que dar ningún tipo de explicación y esto no tendrá ninguna repercusión para mí. Entiendo que el proceso de investigación es independiente de la evaluación de la asignatura Cuidado y Sociedad.

Declaro que he leído la Hoja de Información sobre el estudio citado, se me ha entregado una copia de este Consentimiento Informado, fechado y firmado.

Fecha<br>___/___/__	Nombre Investigador (Mayúsculas)	Firma Investigador

Fecha<br>___/___/___	Nombre Participante (Mayúsculas)	Firma Participante

HOJA DE INFORMACIÓN PARA LOS ESTUDIANTES, PROFESORES E INVESTIGADORES
Fase III - Grupo de Discusión

Invitamos a participar en el estudio de investigación "**Innovación en la comunicación de cuidados paliativos a través de la experiencia docente"** a los estudiantes de la asignatura Cuidado y Sociedad del Instituto Core Curriculum de la Universidad de Navarra.

¿Quiénes somos?
Somos el grupo de investigación ATLANTES, del Instituto Ciencias y Sociedad de la Universidad de Navarra (UNAV), que busca desarrollar una nueva estrategia para la comunicación de los cuidados paliativos a la sociedad.

¿Qué pretendemos?
Nos gustaría contar con su colaboración para participar en un grupo de discusión (GD) con el objetivo de evaluar la asignatura Cuidado y Sociedad.

¿En qué consiste su colaboración?
Su colaboración consiste en participar en un GD con estudiantes y dos facilitadores (investigadores del proyecto), donde se hablará de la experiencia tenida en la asignatura para ajustar actitudes y adquirir y promover información positiva sobre los cuidados paliativos. El GD se realizará en el mes siguiente a la conclusión de la asignatura. El GD será audio grabado y transcrito, para facilitar el análisis de la información. Se enviará un email general a todos los estudiantes conectados a esta asignatura y se seleccionarán los 5 primeros que se apunten a este GD. Cómo incentivo a la participación, se regalará un voucher Amazon de 20€ a los participantes.

Participar en el GD no repercutirá de ninguna forma en la nota de los estudiantes. Garantizamos que tanto el GD como el análisis de los datos obtenidos se llevará a cabo una vez terminada la asignatura cuando las notas ya hayan sido publicadas.

¿Tiene algún coste mi participación?
Participar en el grupo de discusión no tiene ningún costo. Los gastos derivados del desplazamiento (transporte propio o público) para la participación presencial en este grupo serán asumidos por el grupo de investigación (ATLANTES).

Confidencialidad: A todos los que participen en el grupo de discusión se les solicitará confidencialidad de la información. En todo momento se respetará la confidencialidad de sus datos, codificandolos.

a) Confidencialidad y anonimato:
Toda la información que los participantes proporcionen (incluyendo las grabaciones), será tratada

con la más estricta confidencialidad. Los datos se almacenarán durante 3 años en el equipamiento informático de la universidad del investigador principal, de forma anónima, accesibles sólo para el personal de investigación. Toda la información recopilada será confidencial en conformidad con la Ley de protección de datos.

b) Datos personales:

Los datos de este estudio se incorporarán a una base de datos o fichero sin datos personales y el acceso estará disponible al personal del estudio. Se mantendrá una copia de seguridad del archivo de datos personales relacionado con los participantes reclutados, en el disco duro del equipamiento informático de la universidad del investigador principal, accesible al investigador principal. Cada participante recibirá su propio número de identificación de estudio. En todos los resultados del estudio como sean publicaciones o páginas web, sólo se usará datos anonimizados.

Todos los datos personales serán tratados conforme a las leyes actuales de protección de datos, especialmente al Reglamento (UE) 2016/679 del Parlamento Europeo y del Consejo, de 27 de abril de 2016, relativo a la protección de las personas físicas en lo que respecta al tratamiento de datos personales y a la libre circulación de estos, en adelante RGPD.

El Responsable del Tratamiento de los datos de la Universidad de Navarra, en cumplimiento del citado RGDP, le informa que si participa en este estudio sus datos personales serán tratados por el equipo investigador exclusivamente para los fines que usted autorice al firmar la hoja de consentimiento. También podrán acceder a los datos autoridades competentes y los miembros del comité ético, si lo considerasen necesario para supervisar la realización del estudio. No será posible identificarle a usted a través de las comunicaciones que pudiera generar este estudio.

Usted es el responsable de la veracidad y corrección de los datos que nos entrega y tiene la facultad de ejercer los derechos de acceso, rectificación, supresión, limitación del tratamiento, portabilidad y de oposición de sus datos de acuerdo con lo dispuesto en la normativa en materia de protección de datos. Para ejercerlos, deberá dirigirse por escrito al delegado de Protección de Datos de la UN a la siguiente dirección postal Campus Universitario, S/N, Edificio Central. 31080 Pamplona (Navarra, España) o a la dirección de correo electrónico dpo@unav.es, adjuntando una fotocopia de su documento nacional de identidad o equivalente. En caso de no estar de acuerdo con el tratamiento de datos realizado o considerar vulnerados sus derechos, tiene derecho a presentar una reclamación ante la Agencia Española de Protección de datos (www.agpd.es).

Tómese su tiempo antes de decidir sobre su participación y consulte con otras personas si lo desea. Si acepta participar en este grupo de discusión, se le pedirá que rellene unos datos personales como edad, género, nacionalidad, grado que estudia y su firma en el consentimiento informado adjunto a esta información.

Datos de contacto
Le rogamos que lea detenidamente esta información y nos consulte para cualquier duda que pueda tener antes de firmar el consentimiento informado. Para ello, tendrá que ponerse en contacto con Ana Paula Salas o Beatriz Gómez, en la dirección de correo electrónico asalasmo@unav.es o bgomezb@unav.es .

Agradeciendo de antemano su interés y colaboración.

Atentamente,

HOJA DE INFORMACIÓN
Fase III - Grupo de Discusión

Yo (por favor escriba su nombre),

____, he recibido información clara sobre el estudio y acepto participar en el grupo de discusión que tendrá una duración aproximada de 2 horas y que se realizará en la universidad en local y día a designar. Entiendo que la conversación dentro del grupo y que los datos ahí aportados serán tratados de forma confidencial, salvaguardando mi identidad en los informes o publicaciones que se puedan generar con los resultados del estudio. Entiendo también que me es solicitada la confidencialidad de la información que se genere en el grupo de discusión.

Por favor seleccione una opción:

Autorizo participar en este GD
SI ☐ No ☐

Autorizo la grabación de este GD
SI ☐ No ☐

Autorizo su almacenamiento con el resto de datos
SI ☐ No ☐

Autorizo que se guarde mi correo electrónico para ser re-contactado.
SI ☐ No ☐

Declaro que he tenido el tiempo y la oportunidad para realizar preguntas y plantear las dudas que poseyera y que todas las preguntas fueron respondidas a mi entera satisfacción. Entiendo que mi participación es libre y voluntaria y que, si decido no participar más en el estudio en algún momento, no tendré que dar ningún tipo de explicación y esto no tendrá ninguna repercusión para mí. Declaro que he leído la Hoja de Información sobre el estudio citado, se me ha entregado una copia de la Hoja de Información y una copia de este Consentimiento Informado, fechado y firmado

Fecha<br>___/___/___	Nombre Investigador (Mayúsculas)	Firma Investigador
Fecha<br>___/___/___	Nombre Participante (Mayúsculas)	Firma Participante
