# Supplementary material for: Educational innovation as a communication strategy in palliative care: A study protocol and preliminary results
Source: PLoS One. 2023 Jun 9;18(6):e0286343. doi: 10.1371/journal.pone.0286343 (PMC10256175; doi:10.1371/journal.pone.0286343)
Supplement: S2 Protocol — Approved on February 17, 2022. (PDF) [file pone.0286343.s003.pdf]

Ethics committee approved protocol (translated)

Title: "Educational innovation as a communication strategy for palliative care"
Research project of the doctoral thesis of Ana Paula Salas Moreira

1. Definition of objectives and description of the project

1.1 Introduction

Palliative care offers a comprehensive response to improve the quality of life for people facing advanced, incurable and disabling illness (WHO, 2015). However, the term "palliative care" is still associated with the concepts of "dying," "terminal," and "end of life" (Collins, McLachlan, Philip, 2017; Cain, Surbone, Elk, Kagawa-Singer, 2018). New generations maintain a negative and detached view of what palliative care is and what it does, and this way of knowing generates a discourse that has been identified in several studies as a barrier to the implementation of palliative care services in the national health system (Centeno, Garralda, Carrasco, den Herder-van der Eerden, Aldridge, Stevenson, Meier, & Hasselaar, 2017).
In recent years, some social studies have focused on this problem, trying to find a novel way to convey to society the real positive message about PC (McIlfatrick et al., 2021; Reigada, Arantzamendi, & Centeno, 2020; Wallerstedt, Benzein, Schildmeijer, & Sandgren, 2019). The traditional communication that has been developed so far, through media and institutional communication, does not seem to be proving very effective, given the general state of public opinion, and it is therefore necessary to find new ways of communicating.
In a diagnostic study carried out in Spain with university students, it is highlighted that it is positive to count on the students themselves when designing a strategy for explaining and disseminating the palliative care (PC) message (Reigada et al.2021). Therefore, creating an open environment that promotes discussion of the topic among people, through art and creativity, encouraging strategies such as active listening and education for intercultural sensitivity, can help develop an awareness of the process of living with an illness and the convenience of having PC (Reigada et al.2021).
The study by Reigada et al. (2021) already points out that betting on innovative teaching as a public communication strategy may be the right solution to current communication needs. On the other hand, education and communication are two disciplines that, rather than having a close relationship, are inseparable in nature. Education is a space of communication, conditioned by it, possible through it (Hernández, 1996).
In this line, from the Atlantes Reigada et al. (2021) message team, they have studied how to generate knowledge and sensitivity about palliative care among university students. Through Participatory Action Research (PAR), Design Thinking (DT) and adopting a strategy of Public Engagement in Responsible Research and Innovation (PRRI), they have created a social intervention to promote a transformative, active and service learning on palliative care, with and for students of the University of Navarra (UNAV).
This social intervention program becomes a course of the CORE Curriculum Institute of the University of Navarra called "Care and Society" and that will begin in January 2022 with 31 students who are not in health degrees because it is understood that these are the profiles that have less sensitivity to the issue of care but that they can also be the future decision makers, influencers and promoters of an assertive social discourse in society. Through this

social intervention (the course Care and Society) we seek to create ambassadors of the PC message in the UNAV university campus.
The aim of this study is to evaluate the learning experience of students of the course "Care and Society" that seeks to enhance positive attitudes towards care and palliative care of university students.

1.2 Objective
To evaluate the learning experience of students in the course "Care and Society" at the University of Navarra in the academic year 2021/2022.
Specific objectives.
● To evaluate the acceptability and feasibility of the course "Care and Society", as a learning circuit to decrease misinformation and misunderstandings around palliative care, fostering social debate.
● To explore the knowledge and attitudes of university students of the course Care and Society.
● Promote UNAV students to " PC ambassadors" to be the connectors of values around care such as empathy.

2. Methodology
Action-research study, using multiple methods for data collection (such as application of scales, satisfaction questionnaires, artificial intelligence, focus groups and direct observation). Focus group transcripts, field notes, and classroom exercises (e.g., work done by students in each module of the course) will be used for analysis. For each stage of the study, we will detail the methods used for data collection and the respective approaches to data processing (quantitative and qualitative analysis).

2.1 Justification of the selected methods
Action-research is defined as a progressive research method of planning, action, observation, and results to understand and transform socio-educational realities and practices (Lewin, K, 1946). We understand that being a participatory methodology, it will help us to achieve the proposed objectives, assuming a constructivist perspective in this study. That is, the existence of multiple realities can result from human construction where, interactively, it is possible to obtain factual results. Applying this research method that requires a constant analysis of the process, and the intersubjective interpretations that result from there, it is possible to arrive at a valid reality to be explained in a given context (Denzin & Lincoln, 2013).

The overall assessment of the course will focus on four stages:
● Stage 1 - Initial assessment.
● Stage 2 - Assessment of learning.
● Stage 3 - Final assessment
● Stage 4 - Mid-term assessment.

The research questions will be:
1. Is the course Care and Society able to promote students' knowledge about the palliative care and palliative care?
2. Is the course Care and Society able to contribute to students' positive attitudes towards palliative care and palliative care?
palliative care?
3. Is it possible to turn undergraduate students into palliative care ambassadors?

2.2 - Social Intervention Program: Course Care and Society
This course focuses on promoting knowledge, enhancing empathy and positive attitudes of students on issues related to accompanying and caring for people suffering from a serious illness and their environment, in an interdisciplinary way. It is an elective course (3 ECTs) of the Core Curriculum Institute of the University of Navarra.

In the academic year 2021/2022 this course will be offered to a maximum of 30 students from the School of Economics and Business, School of Architecture, School of Education and Psychology. The objective is to evaluate and improve the course to be implemented in the coming years, in UNAV and in other universities in Spain. Therefore, this is considered a pilot study, deeming the course Care and Society the prototype of social intervention.

Module I - Transformative Learning: open to accompany and care
At the end of the module the student will be able to:
● List the situations of serious illness that involve intense suffering;
● Describe when and how to accompany, care and relieve and when not to;
● Outline a set of actions that cooperatively help to care for and alleviate the person in suffering.

Module II - Active learning: the student participates in caregiving.
*Dynamic activity that consists of participating in a self-learning circuit (game "Stay Room") where the students will be the main characters in building their own knowledge related to the topic of caring. At the end of the module the student will be able to:
● Understand what and how is the process of accompanying, caring and alleviating suffering.

Module III - Service Learning: the student as the protagonist of care.
*This module will promote service-learning. Students will participate in community action activities, allowing them to connect what they learn in modules I and II with a real context in an experiential and reflective way. This module will be carried out in collaboration with TANTAKA and the Social Innovation Unit of Navarra. At the end of the module the student will be able to:
● Plan actions based on what they have learned and with a view to developing their personal and professional competencies;
● Evaluate, establish, and recommend good practices in the relationship when caring for people with serious illness.

2.3 - Stages of the evaluation of the social intervention program Care and Society

The evaluation of social intervention programs aims to measure the degree of adequacy, effectiveness and efficiency of the program, as well as to detect and guide improvements to new solutions, facilitating the analysis and prospective design of future interventions. In this study, the evaluation of the intervention will focus on the feasibility and acceptability of the course. Feasibility is understood as the degree of success in terms of implementation and fulfillment of the proposed objectives, and acceptability is reflected through positive feedback from participants.

We will try to evaluate the program in general over time, carrying out a pre-post intervention evaluation. Four evaluation moments will be taken into account: 1) immediately before the beginning of the program (Initial Evaluation), 2) during the program experience (Learning Evaluation) and 3) immediately after the end of the program (Final Evaluation) and 4) 5 months after the end of the course Care and Society (Mid-term Evaluation).

Stage 1 - Initial Assessment (quantitative approach)

1. For the initial evaluation, one week before the start of the course "Care and Society" (2021/2022), students will receive an email inviting them to participate in the study. This email will be sent through the ADI platform. All questionnaires will be done electronically through the Google Forms.

2. The two online questionnaires to be filled in are: one questionnaire that will measure the degree of knowledge about palliative care (Palliative Care knowledge questionnaire) and the other one that measures the level of empathy of the students (Test of Cognitive and Affective Empathy or TECA); the latter has already been acquired by ATLANTES for other studies.

The confidentiality of the data and the anonymity of the students when filling in the questionnaire will be ensured. The following will be requested: "Before starting, please indicate a code for the questionnaire. Indicate the name of a city and three numbers, for example, Pamplona123 (you can NOT use this city, nor this combination of numbers for your code). It is important to remember this code, because it must be used in all the questionnaires we will send you. This will allow us to compare responses without identifying individuals. In addition, it will ensure that the google form does not ask for or display the sender's email address.

4. Without interfering with the academic evaluation of the course professor, at the end of Module I, students who have authorized recontact will be sent links to the second round of the initial palliative care knowledge questionnaire and the TECA.

Instruments: The Palliative Care Knowledge Questionnaire will be developed by the ATLANTES research team, expert in Palliative Care (APPENDIX B); The Cognitive and Affective Empathy Test (TECA) is a global measure of empathy with 33 items, presenting a

four-factor structure to assess: (i) perspective taking regarding the intellectual or imaginative ability to put oneself in another person's place, (ii) the ability to recognize and acknowledge and understand other people's emotions, intentions and impressions, (iii) the ability to share another person's negative emotions, and (iv) the ability to share another person's positive emotions.

Participants:
Students: Students enrolled in the course "Care and Society" of the Core Curriculum 2021/2022 Institute of the University of Navarra.

Stage 2 - Learning Assessment (quantitative and qualitative approach)
The course Care and Society is going to be carried out at the University of Navarra once a week, for two hours, from January to April 2022. In addition to the pre and post intervention evaluation, it is important to carry out continuous evaluations. Therefore, at this stage, students' experiences in each activity will be evaluated. Specifically:

Module I Transformative Learning - This module aims to foster students' self-learning and critical thinking. It is the students who, through small group work and large group discussion, will transmit to others theoretical and practical knowledge on issues related to palliative care. Through case studies provided by the teachers, general discussion and reverse class, at the end of this module students should achieve:
1. Describe the disease situation and how it affects people.
2. Describe the importance of accompanying and caring.
3. Describe a plan of action to accompany and care for a person in a situation of serious illness.

Module II Active Learning - This module offers a dynamic activity, a self-learning circuit where students will be protagonists in building their own knowledge related to the topic of caregiving. There will be a Stay Room (similar to an Escape Room) that has already been prototyped and tested with UNAV students (ethical approval 2020.179). To evaluate the Stay Room experience (Module II), a 10-minute debriefing will be assigned at the end of the game, which will be recorded for teaching purposes. With student consent, transcripts of this recording will be used to help analyze the data.

Students will be asked three questions at the beginning of the session:
1. From 1 to 5, does it indicate the degree of motivation to do the game?
2. From 1 to 5, does it indicate the degree of empathy you consider yourself to have?
3. Tell us about a situation that generates empathy in you.

At the end of the session, we will ask students to respond to these questions:
4. From 1 to 5, indicate whether the game has met your expectation?
5. From 1 to 5, how empathetic do you consider yourself to be?
6. What message do you take away?

Module III Service Learning - In this module it is intended that each student spends a day with a person connected to care (it can be a health professional, a caregiver, a patient).

Knowing that this module is evaluated by the teacher through a written reflection of the students, with the prior consent of the students, this material will be analyzed for research. The students' work should be sent to the researchers by the teachers, who will ensure the anonymity of the authors before submission. Reflections may enhance the values of this experience and, therefore, the written content will be analyzed by two independent researchers, qualitatively and open-ended (thematic analysis/open coding).

Participants:
Students: Students enrolled in the course "Care and Society" of the Core Curriculum 2021/2022 Institute of the University of Navarra.

Stage 3 - Final Evaluation (quantitative and qualitative approach)
Students will receive the last set of questionnaires (final palliative care knowledge and TECA). Using the same participant code selected at the beginning of the study, they will be able to participate in the study by completing the questionnaires online (Google form). They will also be sent a link so that they can give feedback on the course through an interactive satisfaction and commitment questionnaire (developed by the research group), which includes an open question and a commitment to learning in relation to attitude change: "After what you have learned in this course, what do you propose to change in your attitude in the coming months, to improve the palliative care message?". These results will be coded with the same code as the previous questionnaires.
The last two classes of the course contemplate two seminars where students are prepared to argue about palliative care. A social debate will be generated in the classroom on different issues about caregiving, and this debate will be recorded for teaching purposes. The audio of these speeches will be transcribed, ensuring that no data that could identify the students appear, and will be used for research analysis with the prior consent of the students.

Teachers, potential collaborators, and five students of the course will be invited to participate in a discussion group to evaluate the experience. The first five students to respond to this request will be selected by sending an email. The objective of this discussion group is to analyze the strengths and weaknesses of the method, content and other aspects of the course. As a small incentive for participation, a 20€ Amazon voucher will be given to the students. The focus groups will be audio recorded, their content will be transcribed and analyzed by the researchers confidentially.

Participants:
Students: Students enrolled in the course "Care and Society" of the Core Curriculum 2021/2022 Institute of the University of Navarra.

Teachers: Teachers and teaching collaborators of the course "Care and Society" of the Core Curriculum Institute, University of Navarra.

Stage 4 - Mid-term evaluation

Five months after the end of the course, a link to an online questionnaire will be sent to the students by email to indicate the extent to which they have applied the learning commitment. Students will be asked to answer the question "You have made a commitment to change your attitude to improve the palliative care message in the last few months. Have you succeeded in doing so? Please write an example." The same participant code selected at the beginning of the study will be used in this online questionnaire.

3. Analysis

The quantitative analysis will be performed by a researcher with expertise in data analysis from the ATLANTES group. For two qualitative data, a thematic, inductive analysis will be performed without predefined codes of audio transcripts, observation notes, and course materials (e.g., student papers). All data will be analyzed with research questions in mind, which will guide the presentation of the results, showing the rationale and emotional aspects of the participants' experience (thoughts, knowledge, feelings, preconceptions, expectations). The reliability of the research results will be achieved through triangulation (multiple methods, multiple researcher viewpoints, and multiple types of data), which will allow exploring different aspects of the problem and contribute to a consistent interpretation of the data.

4. Research team

**Nombre y rol**	**Perfil**
Ana Paula Salas PhD Student	PhD student at the School of Communication, University of Navarra.
Carla Reigada Thesis advisor	PhD in Psychology. Researcher at ATLANTES, University of Navarra.
Beatriz Gómez Thesis Advisor	PhD in Communication from the School of Communication of the University of Navarra. Professor of written communication and literary journalism.

5. Communication plan

**Activity**	Description	Audience
**Seminar / Workshop**	To hold a morning workshop at the Pamplona City Hall, open by Zoom, on the importance of palliative care and palliative care. The speakers will be members of the research team, and students of the course Care and Society. The conference will be announced through the media.	General Audience

**Press releases and social media**	The dissemination of the study will be carried out through social media, through the profiles of ICS/ATLANTES and the Social Innovation Unit. The ICS website will act as the hub for all information related to the study and where all the contact details of the project team members will be found.<br><br>Write two pieces in well-known newspapers: Diario de Navarra and La Razón.<br><br>Carry out a coverage/small debate with local television (NavarraTV), on the importance of critical thinking in society, in relation to the issue of care and palliative care.	General audeince

**Conferences**	Submit an abstract of the results (oral communication) to a Social Innovation/Education research congress	Academic / Researchers
**Peer-reviewed journals**	Submit at least one scientific article in peer-reviewed journals (Q1/Q2) such as: BMC Palliative Care (IF. 3.815/open Access).	Health professionals / Academic / Researchers
**Seminars**	At least one research seminar that includes different research groups and students, which will take place at UNAV, to present and discuss the study. The speakers will be members of the research group. The conference will be announced through the media.	Health professionals / Academic / Researchers / Students

6. Ethical Considerations

The study will comply at all times with the principles of the European Code of Conduct for Research Integrity. The UNAV Ethics Committee will ensure that the ethical principles of research involving human subjects are respected. Ethical considerations refer to: specific ethical considerations for the three phases of the project; and general considerations for the study as a whole.

7. Appendixes in Spanish (next page)

¡Hola a todos!

Mi nombre es Ana Paula Salas y soy estudiante de la Universidad. Estoy haciendo mi doctorado con el grupo de investigación ATLANTES, y quiero invitarte a formar parte de nuestro estudio "Innovación en la comunicación de cuidados paliativos a través de la experiencia docente".

Lo más importante para mí es **conocer tu experiencia sobre la asignatura "Cuidado y Sociedad"**. Y para hacerlo necesito que los alumnos de la asignatura llenen algunos cuestionarios en **tres momentos** distintos de la asignatura: **antes** de su inicio, **durante** la asignatura, y **después** de finalizadas las clases.

Son cuatro cuestionarios:
1) un **Test de Empatía Cognitiva y Afectiva** (*antes, durante y después*)
2) un cuestionario de **Conocimientos sobre los Cuidados Paliativos inicial** (*antes y durante*)
3) un cuestionario de **Conocimientos sobre los Cuidados Paliativos final** (*después*)
4) un cuestionario de **Satisfacción y compromiso** sobre la asignatura (*después*)

¿Y qué tengo que hacer ahora si decido participar? Es muy sencillo, solamente son **tres pasos** que no te tomarán más de 10 minutos:
Paso 1: Lee la **Hoja de Información** y contáctanos si tienes alguna pregunta.
Paso 2: Rellena estos **DOS cuestionarios** a través de la plataforma Google Forms:
- Conocimientos de cuidados paliativos
- TECA

Paso 3: Para poder enviarte los siguientes pasos del estudio necesito que rellenes esta **autorización de recontacto** a través de este enlace.

Es importante recalcar que **la investigación es independiente a la asignatura.** No interferirá en el desarrollo de las clases, ni repercutirá de ninguna manera en sus notas, ya que es voluntaria. ¡Pero **tu participación es muy valiosa**, y nos sería muy útil para poder mejorar nuestro proyecto!

En la **Hoja de Información** que anexo en este correo podrás encontrar todo lo que necesitas saber sobre nuestro trabajo. No obstante, si tienes alguna duda puedes contactar conmigo (asalas.7@alumni.unav.es) o con Beatriz Gómez (bgomezb@unav.es), quien también es parte del equipo de investigación.

¡Muchas gracias por tu atención! Y recuerda que quedo a tu disposición para cualquier pregunta.

Nos vemos pronto,

Ana Paula Salas

V2.2_ESTUDIANTES_08/02/2022

HOJA DE INFORMACIÓN PARA LOS ESTUDIANTES
Cuestionarios

Te invitamos a participar en el estudio de investigación "Innovación en la comunicación de cuidados paliativos a través de la experiencia docente", a los estudiantes de Asignatura "Cuidado Y Sociedad" del Instituto Core Curriculum de la Universidad de Navarra.

Es importante recalcar que, tanto si decides participar como si no lo haces, no habrá repercusión alguna en la nota de la asignatura ya que el docente no sabrá quién participa, ni las respuestas de quiénes lo hagan. Además, todos los datos recogidos en este estudio solamente serán analizados por el equipo investigador cuando finalice la asignatura y las notas estén publicadas.

Códigos

Con el fin de proteger la identidad de los estudiantes que decidan ayudarnos, al inicio de los cuestionarios cada participante creará su propio código. Este código será utilizado en todos los cuestionarios, de forma que el investigador solo recibirá el Google Form con el código (y sin ningún dato que identifique al estudiante). Así garantizamos que en los resultados solamente se usen datos codificados.

Confidencialidad

A todos los que participen se les pedirá confidencialidad de la información. Los estudiantes recibirán los primeros cuestionarios a través del correo electrónico que a su vez serán enviados a través de la plataforma ADI. El correo electrónico de aquellos estudiantes que decidan participar será guardado por los investigadores para así poder enviarles a los siguientes pasos del estudio. Estos correos electrónicos serán guardados aparte, y no estarán ligados de ninguna manera a las respuestas de los cuestionarios ni a sus códigos.

Las respuestas de los cuestionarios serán analizadas solamente cuando la asignatura esté terminada y las notas lanzadas. De tal forma se garantiza que los docentes no recibirán ningún tipo de información relacionada con el estudio. Los consentimientos informados sobre el contacto se destruirán una vez termine el estudio y no estarán asociados de ninguna manera a las respuestas de los cuestionarios. Toda la información recopilada será confidencial en conformidad con la Ley de protección de datos.

Datos personales

Los datos de este estudio se incorporarán a una base de datos o fichero sin datos personales y el acceso estará disponible al personal del estudio. Se mantendrá una copia de seguridad del archivo de datos personales relacionado con los participantes reclutados, en el disco duro del equipamiento informático de la universidad del investigador principal, accesible al investigador principal durante 3 años.

Todos los datos personales serán tratados conforme a las leyes actuales de protección de datos, especialmente al Reglamento (UE) 2016/679 del Parlamento Europeo y del Consejo, de 27 de abril de 2016, relativo a la protección de las personas físicas en lo que respecta al tratamiento de datos personales y a la libre circulación de estos, en adelante RGPD.

El Responsable del Tratamiento de los datos de la Universidad de Navarra, en cumplimiento del citado RGDP, le informa que si participa en este estudio sus datos personales serán tratados por el equipo investigador exclusivamente para los fines que usted autorice al firmar la hoja de consentimiento. También podrán acceder a los datos autoridades competentes y los miembros del comité ético, si lo considerasen necesario para supervisar la realización del estudio. No será posible identificarle a usted a través de las comunicaciones que pudiera generar este estudio.

Usted es el responsable de la veracidad y corrección de los datos que nos entrega y tiene la facultad de ejercer los derechos de acceso, rectificación, supresión, limitación del tratamiento, portabilidad y de oposición de sus datos de acuerdo con lo dispuesto en la normativa en materia de protección de datos. Para ejercerlos, deberá dirigirse por escrito al delegado de Protección de Datos de la UN a la siguiente dirección postal Campus Universitario, S/N, Edificio Central. 31080 Pamplona (Navarra, España) o a la dirección de correo electrónico dpo@unav.es, adjuntando una fotocopia de su documento nacional de identidad o equivalente. En caso de no estar de acuerdo con el tratamiento de datos realizado o considerar vulnerados sus derechos, tiene derecho a presentar una reclamación ante la Agencia Española de Protección de datos (www.agpd.es).

Tómese su tiempo antes de decidir sobre su participación y consulte con otras personas si lo desea.

Datos de contacto

Somos el grupo de investigación ATLANTES, del Instituto Ciencias y Sociedad de la Universidad de Navarra (UNAV), que busca desarrollar una nueva estrategia para la comunicación de los cuidados paliativos a la sociedad. Le rogamos que lea detenidamente esta información y nos consulte para cualquier duda que pueda tener. Para ello, tendrá que ponerse en contacto con Ana Paula Salas o Beatriz Gómez, en la dirección de correo electrónico asalasmo@unav.es o bgomezb@unav.es.

Agradeciendo de antemano su interés y colaboración.

Atentamente,

Ana Paula Salas

Consentimiento informado para los estudiantes -Recontacto

Estimada/o estudiante de la asignatura Cuidado y Sociedad,

Como ya te contamos, estamos llevando a cabo un estudio titulado "Innovación en la comunicación de cuidados paliativos a través de la experiencia docente", ¡y para sacarlo adelante necesitamos de tu ayuda!

Para seguir analizando la la experiencia del estudiante que cursa la asignatura "Cuidado y Sociedad (2021/2022)" necesitamos que nos des tu correo electrónico y que nos des la autorización para poder enviarte los siguientes pasos de la investigación a través de este medio.

Es importante recalcar que, tanto si decides participar como si no lo haces, no habrá repercusión alguna e[illegible] la nota de la asignatura, ya que el docente no sabrá quién participa, ni las respuestas de quiénes lo hagan. Además, todos los datos recogidos en este estudio solamente serán analizados por el equipo investigador cuando finalice la asignatura y las notas estén publicadas.

¡Muchas gracias por tu colaboración!

*Obligatorio

1. Nombre y Apellido del alumno/a: *

____

2. Mi email: *

____

3. Autorizo que se guarde mi correo electrónico para que los investigadores puedan recontactar conmigo a través de este medio.

Marca solo un óvalo.

- Sí
- No

4. Fecha *

Ejemplo: 7 de enero del 2019

Este contenido no ha sido creado ni aprobado por Google.

Google Formularios

Conocimientos sobre los Cuidados Paliativos

Estimados alumnos/as:

El objetivo del presente cuestionario es evaluar su conocimiento sobre cuidados paliativos. Le agradecemos que exprese con sinceridad sus criterios, pues esta información es completamente confidencial. Para efectos de la evaluación de la asignatura Cuidado y Sociedad, y para efectos de la investigación, le pedimos que rellene el cuestionario antes del curso, después del Módulo I de la asignatura y cuando termine del curso. No le tomará más de 10 minutos.

Este cuestionario forma parte del proyecto "Innovación en la comunicación de cuidados paliativos a través de la experiencia docente", que busca valorar si esta asignatura es una forma eficiente de enviar el mensaje de los cuidados paliativos a la sociedad. Ana Paula Salas es la investigadora principal, y puedes contactar con ella con este correo: asalas.7@alumni.unav.es.

*Obligatorio

1. Antes de iniciar, por favor indique un código para el cuestionario. Indique el nombre de una ciudad y tres números, por ejemplo, Pamplona123 (NO se puede usar esta ciudad ni esta combinación de números para su código). Es importante recordar este código, porque debe s usado en todos los cuestionarios que le enviaremos. Esto nos permitirá comparar respuestas identificar personas. *

____

Cuestionario

Le agradecemos que exprese con sinceridad sus criterios, pues esta información es completamente confidencial.

Proyecto 2021.204
Apobado CEI Unav: Acta 17.02.2022

2. Seleccione los aspectos que caracterizan los Cuidados Paliativos:

Selecciona todos los que correspondan.

- [ ] Dirigidos a disminuir el sufrimiento y elevar la calidad de vida
- [ ] No se deben ofrecer en el hogar
- [ ] Ofrecer apoyo a los pacientes a llevar una vida lo más activa
- [ ] La familia debe participar siempre en los cuidados paliativos
- [ ] Los aspectos no espirituales no son parte de los cuidados paliativos
- [ ] Se ofrecen para retardar la muerte de los pacientes terminales

3. Seleccione las respuestas que le parezcan correctas: "Los cuidados paliativos incluyen a los pacientes..."

Selecciona todos los que correspondan.

- [ ] Con enfermedades no oncológicas crónicas en estadios avanzados
- [ ] Con demencia en estado avanzado
- [ ] Con VIH/SIDA
- [ ] Con Insuficiencia Renal Crónica Terminal
- [ ] Con Enfermedad Pulmonar Obstructiva Crónica (EPOC) o con otra insuficiencia respiratoria
- [ ] Con enfermedades oncológicas en estado terminal
- [ ] Con pronóstico de vida limitado (inferior a 6 meses)

4. Seleccione las respuestas que le parezcan verdaderas: "En los cuidados paliativos se debe ten en cuenta..."

Selecciona todos los que correspondan.

- [ ] Visión integral e individualizada de los pacientes
- [ ] Solo los brindan los médicos de la atención primaria
- [ ] Un médico con conocimientos puede afrontar solo el proceso paliativo
- [ ] Intervenir tanto sobre el paciente como sobre la familia
- [ ] Debe ser atendido por diferentes especialistas según la necesidad
- [ ] Carácter interdisciplinario
- [ ] Pueden realizarse sin preparación previa del equipo de salud
- [ ] Se necesita mucho más que deseos de hacer el bien
- [ ] Intervención en todos los niveles de atención asistenciales
- [ ] Solo importa la voluntad del paciente y no de los cuidadores

5. De las siguientes situaciones seleccione aquellas que sean susceptibles a recibir cuidados paliativos:

Selecciona todos los que correspondan.

- [ ] Varón de 54 años con cáncer de pulmón en fase avanzada, sin expectativas de curación
- [ ] Mujer de 89 años con Insuficiencia Cardiaca avanzada que no mejora pese a tener el tratamiento adecuado
- [ ] Niño de 4 años con enfermedad de generativa en fase avanzada sin tratamiento curativo conocido
- [ ] Ninguno de los anteriores es susceptible de recibir cuidados paliativos

6. Defina en una frase qué son los cuidados paliativos:

Proyecto 2021.204
Apobado CEI Unav: Acta 17.02.2022

7. Fecha:

Ejemplo: 7 de enero del 2019

¡Muchas gracias!

Este contenido no ha sido creado ni aprobado por Google.

Google Formularios

Conocimientos sobre los Cuidados Paliativos (Final)

Estimados alumnos/as:

El objetivo del presente cuestionario es evaluar su conocimiento sobre cuidados paliativos. Le agradecemos que exprese con sinceridad sus criterios, pues esta información es completamente confidencial. Para efectos de la evaluación de la asignatura Cuidado y Sociedad, y para efectos de la investigación, le pedimos que rellene el cuestionario antes del curso, después del Módulo I de la asignatur[illegible] y cuando termine del curso. No le tomará más de 10 minutos.

Este cuestionario forma parte del proyecto "Innovación en la comunicación de cuidados paliativos a través de la experiencia docente", que busca valorar si esta asignatura es una forma eficiente de enviar el mensaje de los cuidados paliativos a la sociedad. Ana Paula Salas es la investigadora principal, y puedes contactar con ella con este correo: asalas.7@alumni.unav.es.

*Obligatorio

1. Por favor escriba el código de su cuestionario (el mismo que creó en el cuestionario inicial): *

____

Cuestionario

Le agradecemos que exprese con sinceridad sus criterios, pues esta información es completamente confidencial.

2. Seleccione los aspectos que caracterizan los Cuidados Paliativos:

Selecciona todos los que correspondan.

- [ ] Dirigidos a disminuir el sufrimiento y elevar la calidad de vida
- [ ] No se deben ofrecer en el hogar
- [ ] Ofrecer apoyo a los pacientes a llevar una vida lo más activa
- [ ] La familia debe participar siempre en los cuidados paliativos
- [ ] Los aspectos no espirituales no son parte de los cuidados paliativos
- [ ] Se ofrecen para retardar la muerte de los pacientes terminales

Proyecto 2021.204
Apobado CEI Unav: Acta 17.02.2022

3. Seleccione las respuestas que le parezcan correctas: "Los cuidados paliativos incluyen a los pacientes..."

Selecciona todos los que correspondan.

- [ ] Con enfermedades no oncológicas crónicas en estadios avanzados
- [ ] Con demencia en estado avanzado
- [ ] Con VIH/SIDA
- [ ] Con Insuficiencia Renal Crónica Terminal
- [ ] Con Enfermedad Pulmonar Obstructiva Crónica (EPOC) o con otra insuficiencia respiratoria
- [ ] Con enfermedades oncológicas en estado terminal
- [ ] Con pronóstico de vida limitado (inferior a 6 meses)

4. Seleccione las respuestas que le parezcan verdaderas: "En los cuidados paliativos se debe ten en cuenta..."

Selecciona todos los que correspondan.

- [ ] Visión integral e individualizada de los pacientes
- [ ] Solo los brindan los médicos de la atención primaria
- [ ] Un médico con conocimientos puede afrontar solo el proceso paliativo
- [ ] Intervenir tanto sobre el paciente como sobre la familia
- [ ] Debe ser atendido por diferentes especialistas según la necesidad
- [ ] Carácter interdisciplinario
- [ ] Pueden realizarse sin preparación previa del equipo de salud
- [ ] Se necesita mucho más que deseos de hacer el bien
- [ ] Intervención en todos los niveles de atención asistenciales
- [ ] Solo importa la voluntad del paciente y no de los cuidadores

5. De las siguientes situaciones seleccione aquellas que sean susceptibles a recibir cuidados paliativos:

Selecciona todos los que correspondan.

- [ ] Varón de 54 años con cáncer de pulmón en fase avanzada, sin expectativas de curación
- [ ] Mujer de 89 años con Insuficiencia Cardiaca avanzada que no mejora pese a tener el tratamiento adecuado
- [ ] Niño de 4 años con enfermedad de generativa en fase avanzada sin tratamiento curativo conocido
- [ ] Ninguno de los anteriores es susceptible de recibir cuidados paliativos

6. Califique la necesidad de formación de futuros profesionales no sanitarios en el área de los cuidados paliativos:

Selecciona todos los que correspondan.

- [ ] Innecesaria
- [ ] Poco necesaria
- [ ] Necesaria
- [ ] Muy necesaria
- [ ] No tengo opinión al respecto

7. ¿Cuál ha sido su experiencia en esta asignatura?

8. ¿Cuál es el mensaje que se lleva de los cuidados paliativos?

9. Fecha:

Ejemplo: 7 de enero del 2019

¡Muchas gracias!

Este contenido no ha sido creado ni aprobado por Google.

Google Formularios

Proyecto 2021.204
Apobado CEI Unav: Acta 17.02.2022

TECA (inicio)

Se han guardado todos los cambios en Drive

Enviar

Preguntas Respuestas Configuración

Escala de la Empatía TECA

Las siguientes frases se refieren a sus sentimientos y pensamientos en distintas situaciones. Indique cómo le describe cada una, eligiendo la puntuación de 1 a 5. Lea cada frase cuidadosamente antes de responder. Conteste honestamente con lo que más se identifique (no hay respuestas correctas o incorrectas). No deje ninguna frase sin contestar.

Para efectos de la evaluación de la asignatura Cuidado y Sociedad, y para efectos de la investigación, le pedimos que rellene el cuestionario antes del curso, después del Módulo I de la asignatura y cuando termine del curso. No le tomará más de 10 minutos.

Este cuestionario forma parte del proyecto "Innovación en la comunicación de cuidados paliativos a través de la experiencia docente", que busca valorar si esta asignatura es una forma eficiente de enviar el mensaje de los cuidados paliativos a la sociedad. Ana Paula Salas es la investigadora principal, y puedes contactar con ella con este correo: asalas.7@alumni.unav.es.

Código del participante. Usa el mismo que usaste en el Cuestionario de Conocimientos (nombre de una ciudad y tres números). *

Texto de respuesta corta

1.- Me resulta fácil darme cuenta de las intenciones de los que me rodean. *

- 1. Totalmente en desacuerdo
- 2. Algo en desacuerdo
- 3. Neutro
- 4. Algo de acuerdo
- 5. Totalmente de acuerdo

2.- Me siento bien si los demás se divierten. *

- 1. Totalmente en desacuerdo
- 2. Algo en desacuerdo
- 3. Neutro
- 4. Algo de acuerdo
- 5. Totalmente de acuerdo

3.- No me pongo triste sólo porque un amigo o amiga lo esté. *

- 1. Totalmente en desacuerdo
- 2. Algo en desacuerdo
- 3. Neutro
- 4. Algo de acuerdo
- 5. Totalmente de acuerdo

Proyecto 2021.204
Apobado CEI Unav: Acta 17.02.2022

4.- Si un amigo o amiga consigue un trabajo muy deseado, me entusiasmo con él o ella. *

- 1. Totalmente en desacuerdo
- 2. Algo en desacuerdo
- 3. Neutro
- 4. Algo de acuerdo
- 5. Totalmente de acuerdo

5.- Me afecta demasiado ver programas de televisión donde se muestran los problemas de otras personas. *

- 1. Totalmente en desacuerdo
- 2. Algo en desacuerdo
- 3. Neutro
- 4. Algo de acuerdo
- 5. Totalmente de acuerdo

6.- Antes de tomar una decisión intento tener en cuenta todos los puntos de vista. *

- 1. Totalmente en desacuerdo
- 2. Algo en desacuerdo
- 3. Neutro
- 4. Algo de acuerdo
- 5. Totalmente de acuerdo

7.- Rara vez reconozco cómo se siente una persona con sólo mirarla. *

- 1. Totalmente en desacuerdo
- 2. Algo en desacuerdo
- 3. Neutro
- 4. Algo de acuerdo
- 5. Totalmente de acuerdo

8.- Me afecta poco escuchar desgracias sobre personas desconocidas. *

- 1. Totalmente en desacuerdo
- 2. Algo en desacuerdo
- 3. Neutro
- 4. Algo de acuerdo
- 5. Totalmente de acuerdo

9.- Me alegra ver que alguien nuevo se encuentra a gusto en nuestro grupo. *

- 1. Totalmente en desacuerdo
- 2. Algo en desacuerdo
- 3. Neutro
- 4. Algo de acuerdo
- 5. Totalmente de acuerdo

Proyecto 2021.204
Apobado CEI Unav: Acta 17.02.2022

10.- Me es difícil entender cómo se siente una persona ante una situación que no he vivido. *

1. Totalmente en desacuerdo
2. Algo en desacuerdo
3. Neutro
4. Algo de acuerdo
5. Totalmente de acuerdo

11.- Cuando alguien cercano se ha portado mal conmigo intento entender sus motivos. *

1. Totalmente en desacuerdo
2. Algo en desacuerdo
3. Neutro
4. Algo de acuerdo
5. Totalmente de acuerdo

Proyecto 2021.204
Apobado CEI Unav: Acta 17.02.2022

12.- Menos que se trate de algo muy grave, me cuesta llorar con lo que les sucede a otros. *

- 1. Totalmente en desacuerdo
- 2. Algo en desacuerdo
- 3. Neutro
- 4. Algo de acuerdo
- 5. Totalmente de acuerdo

13.- Reconozco fácilmente cuando alguien está de mal humor. *

- 1. Totalmente en desacuerdo
- 2. Algo en desacuerdo
- 3. Neutro
- 4. Algo de acuerdo
- 5. Totalmente de acuerdo

14.- No siempre me doy cuenta cuando la persona que tengo al lado se siente mal. *

- 1. Totalmente en desacuerdo
- 2. Algo en desacuerdo
- 3. Neutro
- 4. Algo de acuerdo
- 5. Totalmente de acuerdo

15.- Intento ponerme en el lugar de los demás para saber cómo actuarán. *

- 1. Totalmente en desacuerdo
- 2. Algo en desacuerdo
- 3. Neutro
- 4. Algo de acuerdo
- 5. Totalmente de acuerdo

16.- Cuando a alguien le sucede algo bueno, siento alegría. *

- 1. Totalmente en desacuerdo
- 2. Algo en desacuerdo
- 3. Neutro
- 4. Algo de acuerdo
- 5. Totalmente de acuerdo

17.- Cuando tengo una opinión formada, no presto mucha atención a los argumentos de los demás. *

- 1. Totalmente en desacuerdo
- 2. Algo en desacuerdo
- 3. Neutro
- 4. Algo de acuerdo
- 5. Totalmente de acuerdo

18.- A veces sufro más con las desgracias de otras personas que ellas mismas. *

- 1. Totalmente en desacuerdo
- 2. Algo en desacuerdo
- 3. Neutro
- 4. Algo de acuerdo
- 5. Totalmente de acuerdo

19.- Me siento feliz sólo con ver felices a otras personas. *

- 1. Totalmente en desacuerdo
- 2. Algo en desacuerdo
- 3. Neutro
- 4. Algo de acuerdo
- 5. Totalmente de acuerdo

20.- Cuando alguien tiene un problema intento imaginarme cómo me sentiría si estuviera en su situación. *

- 1. Totalmente en desacuerdo
- 2. Algo en desacuerdo
- 3. Neutro
- 4. Algo de acuerdo
- 5. Totalmente de acuerdo

21.- No siento especial alegría si alguien me cuenta que ha tenido un golpe de suerte. *

- 1. Totalmente en desacuerdo
- 2. Algo en desacuerdo
- 3. Neutro
- 4. Algo de acuerdo
- 5. Totalmente de acuerdo

22.- Cuando veo que alguien recibe un regalo, no puedo reprimir una sonrisa. *

- 1. Totalmente en desacuerdo
- 2. Algo en desacuerdo
- 3. Neutro
- 4. Algo de acuerdo
- 5. Totalmente de acuerdo

23.- No puedo evitar llorar con los testimonios de personas desconocidas. *

- 1. Totalmente en desacuerdo
- 2. Algo en desacuerdo
- 3. Neutro
- 4. Algo de acuerdo
- 5. Totalmente de acuerdo

24.- Cuando conozco gente nueva me doy cuenta de la impresión que se han llevado de mi. *

- 1. Totalmente en desacuerdo
- 2. Algo en desacuerdo
- 3. Neutro
- 4. Algo de acuerdo
- 5. Totalmente de acuerdo

25.- Cuando mis amigos o amigas me cuentan que les va bien, no le doy mucha importancia. *

- 1. Totalmente en desacuerdo
- 2. Algo en desacuerdo
- 3. Neutro
- 4. Algo de acuerdo
- 5. Totalmente de acuerdo

26.- Encuentro difícil ver las cosas desde el punto de vista de otras personas. *

- 1. Totalmente en desacuerdo
- 2. Algo en desacuerdo
- 3. Neutro
- 4. Algo de acuerdo
- 5. Totalmente de acuerdo

27.- Entender cómo se siente otra persona es algo muy fácil para mí. *

- 1. Totalmente en desacuerdo
- 2. Algo en desacuerdo
- 3. Neutro
- 4. Algo de acuerdo
- 5. Totalmente de acuerdo

Proyecto 2021.204
Apobado CEI Unav: Acta 17.02.2022

28.- No soy de esas personas que se deprimen con los problemas de los demás. *

- 1. Totalmente en desacuerdo
- 2. Algo en desacuerdo
- 3. Neutro
- 4. Algo de acuerdo
- 5. Totalmente de acuerdo

29.- Intento comprender mejor a mis amigos y amigas mirando las situaciones desde su perspectiva. *

- 1. Totalmente en desacuerdo
- 2. Algo en desacuerdo
- 3. Neutro
- 4. Algo de acuerdo
- 5. Totalmente de acuerdo

30.- Me considero una persona fría porque no me conmuevo fácilmente. *

- 1. Totalmente en desacuerdo
- 2. Algo en desacuerdo
- 3. Neutro
- 4. Algo de acuerdo
- 5. Totalmente de acuerdo

31.- Me doy cuenta cuando las personas cercanas a mí están especialmente contentas sin que me hayan contado el motivo. *

- 1. Totalmente en desacuerdo
- 2. Algo en desacuerdo
- 3. Neutro
- 4. Algo de acuerdo
- 5. Totalmente de acuerdo

32.- Me resulta difícil ponerme en el lugar de personas con las que no estoy de acuerdo. *

- 1. Totalmente en desacuerdo
- 2. Algo en desacuerdo
- 3. Neutro
- 4. Algo de acuerdo
- 5. Totalmente de acuerdo

33.- Me doy cuenta cuando alguien intenta esconder sus verdaderos sentimientos. *

- 1. Totalmente en desacuerdo
- 2. Algo en desacuerdo
- 3. Neutro
- 4. Algo de acuerdo
- 5. Totalmente de acuerdo

Valoración de la asignatura Cuidado y Sociedad

Por favor, valora la experiencia y déjanos alguna recomendación para mejorar. Muchas gracias por tu tiempo.

1. Código de participante (el mismo usado en los cuestionarios: ciudad + 3 números)

____

2. Valora la experiencia de esta asignatura:

Marca solo un óvalo.

	1	2	3	4	5	
Muy mala	◯	◯	◯	◯	◯	Muy buena

3. Valora la metodología de enseñanza:

Marca solo un óvalo.

	1	2	3	4	5	
Muy mala	◯	◯	◯	◯	◯	Muy buena

4. Menciona una de las cosas más importantes que hayas aprendido:

5. Cómo crees que se podría mejorar la asignatura:

6. Después lo que has aprendido, ¿qué te propones cambiar en tu actitud en los próximos meses para mejorar el mensaje de los cuidados paliativos?

Este contenido no ha sido creado ni aprobado por Google.

Google Formularios

HOJA DE INFORMACIÓN PARA LOS ESTUDIANTES
Material de Clase

Te invitamos a participar en el estudio de investigación "Innovación en la comunicación de cuidados paliativos a través de la experiencia docente", a los estudiantes de asignatura "Cuidado y Sociedad" del Instituto Core Curriculum de la Universidad de Navarra.

Es importante recalcar que, tanto si decides participar como si no lo haces, no habrá repercusión alguna en la asignatura. El docente no sabrá quién participa, ni las respuestas de quiénes lo hagan. Además, todos los datos recogidos en este estudio solamente serán analizados por el equipo investigador cuando finalice la asignatura y las notas estén publicadas.

¿En qué consiste su colaboración?
Queremos que **nos compartas tu experiencia sobre la asignatura "Cuidado y Sociedad (2021/2022)"**. Para ello, nos gustaría que nos dieras tu consentimiento para acceder a los siguientes materiales de clase: los trabajos escritos y las transcripciones de audio.

1) **Trabajos escritos:** Tu colaboración consiste en **permitir que tus trabajos escritos en clase sean utilizados para la investigación**, asegurando que tu nombre no esté asociado de ninguna manera. Se le pedirá a los docentes que nos entreguen el material sin ningún dato que identifique a los alumnos.

2) **Transcripciones de audio:** Durante la asignatura, ciertas actividades (debriefing posterior a la dinámica del "Stay Room" y el discurso social creado en clase para el módulo III) fueron grabadas con fines de la docencia. Estas grabaciones han sido transcritas por un docente asegurando que no contienen ningún dato personal de los alumnos. Tu colaboración consiste en **permitir que tengamos acceso a esas transcripciones**.

Confidencialidad
A todos los que participen se les pedirá confidencialidad de la información. En todo momento se respetará la confidencialidad de sus datos. Estos materiales no estarán asociadas al nombre de los alumnos, ni estarán enlazados de ninguna manera a los códigos de los cuestionarios. Por lo tanto, los resultados serán siempre publicados garantizando el anonimato.

Este consentimiento informado se recogerá el último día de clases, y permanecerá en un sobre cerrado hasta que la asignatura finalice y las notas sean publicadas. Los consentimientos informados no se guardarán. Se destruirán una vez termine la asignatura. Toda la información recopilada será confidencial en conformidad con la Ley de protección de datos.

Datos personales
Los datos de este estudio se incorporarán a una base de datos o fichero sin datos personales y el acceso estará disponible al personal del estudio. Se mantendrá una copia de seguridad del archivo de datos personales relacionado con los participantes reclutados, en el disco duro del equipamiento informático de la universidad del investigador principal, accesible al investigador principal durante 3 años.

Todos los datos personales serán tratados conforme a las leyes actuales de protección de datos, especialmente al Reglamento (UE) 2016/679 del Parlamento Europeo y del Consejo, de 27 de abril de 2016, relativo a la protección de las personas físicas en lo que respecta al tratamiento de datos personales y a la libre circulación de estos, en adelante RGPD.

El Responsable del Tratamiento de los datos de la Universidad de Navarra, en cumplimiento del

citado RGDP, le informa que si participa en este estudio sus datos personales serán tratados por el equipo investigador exclusivamente para los fines que usted autorice al firmar la hoja de consentimiento. También podrán acceder a los datos autoridades competentes y los miembros del comité ético, si lo considerasen necesario para supervisar la realización del estudio. No será posible identificarle a usted a través de las comunicaciones que pudiera generar este estudio.

Usted es el responsable de la veracidad y corrección de los datos que nos entrega y tiene la facultad de ejercer los derechos de acceso, rectificación, supresión, limitación del tratamiento, portabilidad y de oposición de sus datos de acuerdo con lo dispuesto en la normativa en materia de protección de datos. Para ejercerlos, deberá dirigirse por escrito al delegado de Protección de Datos de la UN a la siguiente dirección postal Campus Universitario, S/N, Edificio Central. 31080 Pamplona (Navarra, España) o a la dirección de correo electrónico dpo@unav.es, adjuntando una fotocopia de su documento nacional de identidad o equivalente. En caso de no estar de acuerdo con el tratamiento de datos realizado o considerar vulnerados sus derechos, tiene derecho a presentar una reclamación ante la Agencia Española de Protección de datos (www.agpd.es).

Tómese su tiempo antes de decidir sobre su participación y consulte con otras personas si lo desea. Si acepta participar en este grupo de diseño, se le pedirá que rellene unos datos personales como edad, género, nacionalidad, grado que cursa y su firma en el consentimiento informado adjunto a esta información.

Datos de contacto

Somos el grupo de investigación ATLANTES, del Instituto Ciencias y Sociedad de la Universidad de Navarra (UNAV), que busca desarrollar una nueva estrategia para la comunicación de los cuidados paliativos a la sociedad. Le rogamos que lea detenidamente esta información y nos consulte para cualquier duda que pueda tener antes de firmar el consentimiento informado. Para ello, tendrá que ponerse en contacto con Ana Paula Salas o Beatriz Gómez, en la dirección de correo electrónico asalasmo@unav.es o bgomezb@unav.es.

Agradeciendo de antemano su interés y colaboración.

Atentamente,

Ana Paula Salas

CONSENTIMIENTO INFORMADO PARA LOS ESTUDIANTES
Material de clase

Yo (por favor escriba su nombre),

____, he recibido información clara sobre el estudio que lleva por título **Innovación en la comunicación de cuidados paliativos a través de la experiencia docente** y acepto participar. Entiendo que los datos aportados serán tratados de forma confidencial, salvaguardando mi identidad en los informes o publicaciones que se puedan generar con los resultados del estudio. Entiendo también que me es solicitada la confidencialidad de la información que se genere en la asignatura.

Por favor seleccione una opción:

Autorizo que se utilicen los trabajos escritos producidos en clase, garantizando que no estarán asociados de ninguna manera a datos que puedan identificar al estudiante.

SI ___ No ___

Autorizo que se utilicen las transcripciones de las grabaciones de clase, garantizando que se eliminará cualquier información que identifique a estudiantes.

SI ___ No ___

Declaro que he tenido el tiempo y la oportunidad para realizar preguntas y plantear las dudas que poseyera, y que todas las preguntas fueron respondidas a mi entera satisfacción.

Entiendo que mi participación es libre y voluntaria y que, si decido no participar más en el estudio en algún momento, no tendré que dar ningún tipo de explicación y esto no tendrá ninguna repercusión para mí. Entiendo que el proceso de investigación es independiente de la evaluación de la asignatura Cuidado y Sociedad.

Declaro que he leído la Hoja de Información sobre el estudio citado, se me ha entregado una copia de este Consentimiento Informado, fechado y firmado.

Fecha<br>___/___/__	Nombre Investigador (Mayúsculas)	Firma Investigador

Fecha<br>___/___/___	Nombre Participante (Mayúsculas)	Firma Participante

HOJA DE INFORMACIÓN PARA LOS ESTUDIANTES, PROFESORES E INVESTIGADORES
Fase III - Grupo de Discusión

Invitamos a participar en el estudio de investigación "**Innovación en la comunicación de cuidados paliativos a través de la experiencia docente"** a los estudiantes de la asignatura Cuidado y Sociedad del Instituto Core Curriculum de la Universidad de Navarra.

¿Quiénes somos?
Somos el grupo de investigación ATLANTES, del Instituto Ciencias y Sociedad de la Universidad de Navarra (UNAV), que busca desarrollar una nueva estrategia para la comunicación de los cuidados paliativos a la sociedad.

¿Qué pretendemos?
Nos gustaría contar con su colaboración para participar en un grupo de discusión (GD) con el objetivo de evaluar la asignatura Cuidado y Sociedad.

¿En qué consiste su colaboración?
Su colaboración consiste en participar en un GD con estudiantes y dos facilitadores (investigadores del proyecto), donde se hablará de la experiencia tenida en la asignatura para ajustar actitudes y adquirir y promover información positiva sobre los cuidados paliativos. El GD se realizará en el mes siguiente a la conclusión de la asignatura. El GD será audio grabado y transcrito, para facilitar el análisis de la información. Se enviará un email general a todos los estudiantes conectados a esta asignatura y se seleccionarán los 5 primeros que se apunten a este GD. Cómo incentivo a la participación, se regalará un voucher Amazon de 20€ a los participantes.

Participar en el GD no repercutirá de ninguna forma en la nota de los estudiantes. Garantizamos que tanto el GD como el análisis de los datos obtenidos se llevará a cabo una vez terminada la asignatura cuando las notas ya hayan sido publicadas.

¿Tiene algún coste mi participación?
Participar en el grupo de discusión no tiene ningún costo. Los gastos derivados del desplazamiento (transporte propio o público) para la participación presencial en este grupo serán asumidos por el grupo de investigación (ATLANTES).

Confidencialidad: A todos los que participen en el grupo de discusión se les solicitará confidencialidad de la información. En todo momento se respetará la confidencialidad de sus datos, codificandolos.

a) Confidencialidad y anonimato:
Toda la información que los participantes proporcionen (incluyendo las grabaciones), será tratada

con la más estricta confidencialidad. Los datos se almacenarán durante 3 años en el equipamiento informático de la universidad del investigador principal, de forma anónima, accesibles sólo para el personal de investigación. Toda la información recopilada será confidencial en conformidad con la Ley de protección de datos.

b) Datos personales:

Los datos de este estudio se incorporarán a una base de datos o fichero sin datos personales y el acceso estará disponible al personal del estudio. Se mantendrá una copia de seguridad del archivo de datos personales relacionado con los participantes reclutados, en el disco duro del equipamiento informático de la universidad del investigador principal, accesible al investigador principal. Cada participante recibirá su propio número de identificación de estudio. En todos los resultados del estudio como sean publicaciones o páginas web, sólo se usará datos anonimizados.

Todos los datos personales serán tratados conforme a las leyes actuales de protección de datos, especialmente al Reglamento (UE) 2016/679 del Parlamento Europeo y del Consejo, de 27 de abril de 2016, relativo a la protección de las personas físicas en lo que respecta al tratamiento de datos personales y a la libre circulación de estos, en adelante RGPD.

El Responsable del Tratamiento de los datos de la Universidad de Navarra, en cumplimiento del citado RGDP, le informa que si participa en este estudio sus datos personales serán tratados por el equipo investigador exclusivamente para los fines que usted autorice al firmar la hoja de consentimiento. También podrán acceder a los datos autoridades competentes y los miembros del comité ético, si lo considerasen necesario para supervisar la realización del estudio. No será posible identificarle a usted a través de las comunicaciones que pudiera generar este estudio.

Usted es el responsable de la veracidad y corrección de los datos que nos entrega y tiene la facultad de ejercer los derechos de acceso, rectificación, supresión, limitación del tratamiento, portabilidad y de oposición de sus datos de acuerdo con lo dispuesto en la normativa en materia de protección de datos. Para ejercerlos, deberá dirigirse por escrito al delegado de Protección de Datos de la UN a la siguiente dirección postal Campus Universitario, S/N, Edificio Central. 31080 Pamplona (Navarra, España) o a la dirección de correo electrónico dpo@unav.es, adjuntando una fotocopia de su documento nacional de identidad o equivalente. En caso de no estar de acuerdo con el tratamiento de datos realizado o considerar vulnerados sus derechos, tiene derecho a presentar una reclamación ante la Agencia Española de Protección de datos (www.agpd.es).

Tómese su tiempo antes de decidir sobre su participación y consulte con otras personas si lo desea. Si acepta participar en este grupo de discusión, se le pedirá que rellene unos datos personales como edad, género, nacionalidad, grado que estudia y su firma en el consentimiento informado adjunto a esta información.

Datos de contacto

Le rogamos que lea detenidamente esta información y nos consulte para cualquier duda que pueda tener antes de firmar el consentimiento informado. Para ello, tendrá que ponerse en contacto con Ana Paula Salas o Beatriz Gómez, en la dirección de correo electrónico asalasmo@unav.es o bgomezb@unav.es .

Agradeciendo de antemano su interés y colaboración.

Atentamente,

HOJA DE INFORMACIÓN
Fase III - Grupo de Discusión

Yo (por favor escriba su nombre),

____, he recibido información clara sobre el estudio y acepto participar en el grupo de discusión que tendrá una duración aproximada de 2 horas y que se realizará en la universidad en local y día a designar. Entiendo que la conversación dentro del grupo y que los datos ahí aportados serán tratados de forma confidencial, salvaguardando mi identidad en los informes o publicaciones que se puedan generar con los resultados del estudio. Entiendo también que me es solicitada la confidencialidad de la información que se genere en el grupo de discusión.

Por favor seleccione una opción:

Autorizo participar en este GD
SI ☐ No ☐

Autorizo la grabación de este GD
SI ☐ No ☐

Autorizo su almacenamiento con el resto de datos
SI ☐ No ☐

Autorizo que se guarde mi correo electrónico para ser re-contactado.
SI ☐ No ☐

Declaro que he tenido el tiempo y la oportunidad para realizar preguntas y plantear las dudas que poseyera y que todas las preguntas fueron respondidas a mi entera satisfacción. Entiendo que mi participación es libre y voluntaria y que, si decido no participar más en el estudio en algún momento, no tendré que dar ningún tipo de explicación y esto no tendrá ninguna repercusión para mí. Declaro que he leído la Hoja de Información sobre el estudio citado, se me ha entregado una copia de la Hoja de Información y una copia de este Consentimiento Informado, fechado y firmado

Fecha<br>___/___/___	Nombre Investigador (Mayúsculas)	Firma Investigador
Fecha<br>___/___/___	Nombre Participante (Mayúsculas)	Firma Participante
