# Supplementary material for: Educational innovation as a communication strategy in palliative care: A study protocol and preliminary results
Source: PLoS One. 2023 Jun 9;18(6):e0286343. doi: 10.1371/journal.pone.0286343 (PMC10256175; doi:10.1371/journal.pone.0286343)
Supplement: S1 File — Answers with codes that appear in at least two moments of evaluation were included. Answers with codes that appear only in one moment of evaluation or answers with repeated codes in the same moment of evaluation were excluded. (PDF) [file pone.0286343.s004.pdf]

Repetición	Fecha	Código	Seleccione los aspectos que caracterizan los Cuidados Paliativos:	Seleccione las respuestas que le parezcan correctas: "Los cuidados paliativos incluyen a los pacientes..."	Seleccione las respuestas que le parezcan verdaderas: "En los cuidados paliativos se debe tener en cuenta..."	De las siguientes situaciones seleccione aquellas que sean susceptibles a recibir cuidados paliativos:	Defina en una frase qué son los cuidados paliativos:<br>o<br>Califique la necesidad de formación de futuros profesionales no sanitarios en el área de los cuidados paliativos:	¿Cuál ha sido su experiencia en esta asignatura? (M3)	¿Cuál es el mensaje que se lleva de los cuidados paliativos?
En los 3 momentos de evaluación	12/01/2022	Paris358	Dirigidos a disminuir el sufrimiento y elevar la calidad de vida	Con enfermedades no oncológicas crónicas en estadios avanzados, Con enfermedades oncológicas en estado terminal, Con pronóstico de vida limitado (inferior a 6 meses)	Visión integral e individualizada de los pacientes, Intervenir tanto sobre el paciente como sobre la familia, Debe ser atendido por diferentes especialistas según la necesidad, Carácter interdisciplinario, Se necesita mucho más que deseos de hacer el bien, Intervención en todos los niveles de atención asistenciales	Varón de 54 años con cáncer de pulmón en fase avanzada, sin expectativas de curación, Mujer de 89 años con Insuficiencia Cardiaca avanzada que no mejora pese a tener el tratamiento adecuado, Niño de 4 años con enfermedad de generativa en fase avanzada sin tratamiento curativo conocido	Aquella atención y apoyo que reciben los pacientes y sus familias en situación de enfermedad terminal.	-	-
	01/03/2022	Paris358	Dirigidos a disminuir el sufrimiento y elevar la calidad de vida	Con enfermedades no oncológicas crónicas en estadios avanzados, Con demencia en estado avanzado, Con Insuficiencia Renal Crónica Terminal, Con enfermedades oncológicas en estado terminal, Con pronóstico de vida limitado (inferior a 6 meses)	Visión integral e individualizada de los pacientes, Intervenir tanto sobre el paciente como sobre la familia, Debe ser atendido por diferentes especialistas según la necesidad, Carácter interdisciplinario, Se necesita mucho más que deseos de hacer el bien, Intervención en todos los niveles de atención asistenciales	Varón de 54 años con cáncer de pulmón en fase avanzada, sin expectativas de curación, Niño de 4 años con enfermedad de generativa en fase avanzada sin tratamiento curativo conocido	Los cuidados paliativos son el apoyo, ayuda y asistencia dada a los pacientes en enfermedades avanzadas con expectativa de muerte próxima, para que tengan una mejor calidad de vida y comodidad y se reduzca su sufrimiento.	-	-
	28/04/2022	Paris358	Dirigidos a disminuir el sufrimiento y elevar la calidad de vida, La familia debe participar siempre en los cuidados paliativos, +apoyo espiritual y social	Con enfermedades no oncológicas crónicas en estadios avanzados, Con Insuficiencia Renal Crónica Terminal, Con Enfermedad Pulmonar Obstructiva Crónica (EPOC) o con otra insuficiencia respiratoria, Con enfermedades oncológicas en estado terminal, Con pronóstico de vida limitado (inferior a 6 meses)	Visión integral e individualizada de los pacientes, Intervenir tanto sobre el paciente como sobre la familia, Debe ser atendido por diferentes especialistas según la necesidad, Carácter interdisciplinario, Se necesita mucho más que deseos de hacer el bien, Intervención en todos los niveles de atención asistenciales	Varón de 54 años con cáncer de pulmón en fase avanzada, sin expectativas de curación, Mujer de 89 años con Insuficiencia Cardiaca avanzada que no mejora pese a tener el tratamiento adecuado, Niño de 4 años con enfermedad de generativa en fase avanzada sin tratamiento curativo conocido	Muy necesaria	Lo he disfrutado mucho y he aprendido lo que son los cuidados paliativos y su realidad	Que todos valemos por igual hasta el último día y que merecemos paz, cuidado y bienestar
	19/01/2022	Bilbao842	Dirigidos a disminuir el sufrimiento y elevar la calidad de vida, La familia debe participar siempre en los cuidados paliativos	Con enfermedades no oncológicas crónicas en estadios avanzados, Con VIH/SIDA, Con Insuficiencia Renal Crónica Terminal, Con Enfermedad Pulmonar Obstructiva Crónica (EPOC) o con otra insuficiencia respiratoria, Con enfermedades oncológicas en estado terminal, Con pronóstico de vida limitado (inferior a 6 meses)	Visión integral e individualizada de los pacientes, Intervenir tanto sobre el paciente como sobre la familia, Debe ser atendido por diferentes especialistas según la necesidad, Carácter interdisciplinario, Se necesita mucho más que deseos de hacer el bien, Intervención en todos los niveles de atención asistenciales	Varón de 54 años con cáncer de pulmón en fase avanzada, sin expectativas de curación, Mujer de 89 años con Insuficiencia Cardiaca avanzada que no mejora pese a tener el tratamiento adecuado, Niño de 4 años con enfermedad de generativa en fase avanzada sin tratamiento curativo conocido	El acompañamiento y cuidado del cuerpo y alma del paciente y su familia	-	-
	25/03/2022	Bilbao842	Dirigidos a disminuir el sufrimiento y elevar la calidad de vida, La familia debe participar siempre en los cuidados paliativos	Con enfermedades no oncológicas crónicas en estadios avanzados, Con demencia en estado avanzado, Con VIH/SIDA, Con Insuficiencia Renal Crónica Terminal, Con Enfermedad Pulmonar Obstructiva Crónica (EPOC) o con otra insuficiencia respiratoria, Con enfermedades oncológicas en estado terminal, Con pronóstico de vida limitado (inferior a 6 meses)	Visión integral e individualizada de los pacientes, Intervenir tanto sobre el paciente como sobre la familia, Debe ser atendido por diferentes especialistas según la necesidad, Carácter interdisciplinario, Se necesita mucho más que deseos de hacer el bien, Intervención en todos los niveles de atención asistenciales	Varón de 54 años con cáncer de pulmón en fase avanzada, sin expectativas de curación, Mujer de 89 años con Insuficiencia Cardiaca avanzada que no mejora pese a tener el tratamiento adecuado, Niño de 4 años con enfermedad de generativa en fase avanzada sin tratamiento curativo conocido	Acompañamiento y cuidado en enfermedad grave con dolor agudo, alivio del sufrimiento en todas las dimensiones del ser humano	-	-
	28/04/2022	Bilbao842	Dirigidos a disminuir el sufrimiento y elevar la calidad de vida, La familia debe participar siempre en los cuidados paliativos	Con enfermedades no oncológicas crónicas en estadios avanzados, Con demencia en estado avanzado, Con VIH/SIDA, Con Insuficiencia Renal Crónica Terminal, Con Enfermedad Pulmonar Obstructiva Crónica (EPOC) o con otra insuficiencia respiratoria, Con enfermedades oncológicas en estado terminal, Con pronóstico de vida limitado (inferior a 6 meses)	Visión integral e individualizada de los pacientes, Intervenir tanto sobre el paciente como sobre la familia, Debe ser atendido por diferentes especialistas según la necesidad, Carácter interdisciplinario, Intervención en todos los niveles de atención asistenciales	Varón de 54 años con cáncer de pulmón en fase avanzada, sin expectativas de curación, Mujer de 89 años con Insuficiencia Cardiaca avanzada que no mejora pese a tener el tratamiento adecuado, Niño de 4 años con enfermedad de generativa en fase avanzada sin tratamiento curativo conocido	Necesaria	Estoy muy contenta con la asignatura y su planteamiento con los distintos módulos. Creo que va a ser muy beneficiosa para todos los alumnos que tengan la suerte de cursarla.	Que todos debemos formarnos en el buen cuidado porque todos merecemos ser bien cuidados hasta el final
	20/01/2022	pamplona111	Ofrecer apoyo a los pacientes a llevar una vida lo más activa, La familia debe participar siempre en los cuidados paliativos	Con enfermedades no oncológicas crónicas en estadios avanzados, Con enfermedades oncológicas en estado terminal, Con pronóstico de vida limitado (inferior a 6 meses)	Visión integral e individualizada de los pacientes, Intervenir tanto sobre el paciente como sobre la familia, Debe ser atendido por diferentes especialistas según la necesidad, Carácter interdisciplinario, Intervención en todos los niveles de atención asistenciales	Varón de 54 años con cáncer de pulmón en fase avanzada, sin expectativas de curación, Niño de 4 años con enfermedad de generativa en fase avanzada sin tratamiento curativo conocido	Acompañamiento a personas en estado terminal	-	-
	25/03/2022	pamplona111	Dirigidos a disminuir el sufrimiento y elevar la calidad de vida, La familia debe participar siempre en los cuidados paliativos	Con demencia en estado avanzado, Con enfermedades oncológicas en estado terminal	Visión integral e individualizada de los pacientes, Debe ser atendido por diferentes especialistas según la necesidad, Intervención en todos los niveles de atención asistenciales	Varón de 54 años con cáncer de pulmón en fase avanzada, sin expectativas de curación, Niño de 4 años con enfermedad de generativa en fase avanzada sin tratamiento curativo conocido	Cuidar para hacer menor el sufrimiento de una persona que esta enferma terminal	-	-
	28/04/2022	pamplona111	Dirigidos a disminuir el sufrimiento y elevar la calidad de vida, No se deben ofrecer en el hogar, Ofrecer apoyo a los pacientes a llevar una vida lo más activa	Con enfermedades no oncológicas crónicas en estadios avanzados, Con demencia en estado avanzado, Con Insuficiencia Renal Crónica Terminal, Con pronóstico de vida limitado (inferior a 6 meses)	Intervenir tanto sobre el paciente como sobre la familia, Debe ser atendido por diferentes especialistas según la necesidad, Se necesita mucho más que deseos de hacer el bien, Intervención en todos los niveles de atención asistenciales	Varón de 54 años con cáncer de pulmón en fase avanzada, sin expectativas de curación, Mujer de 89 años con Insuficiencia Cardiaca avanzada que no mejora pese a tener el tratamiento adecuado, Niño de 4 años con enfermedad de generativa en fase avanzada sin tratamiento curativo conocido	Necesaria	Me ha ayudado a enterarme de la dura realidad de estas situaciones	Que todos merecemos amor y que todos necesitamos dar amor
	03/02/2022	Turín222	La familia debe participar siempre en los cuidados paliativos, Se ofrecen para retardar la muerte de los pacientes terminales	Con enfermedades no oncológicas crónicas en estadios avanzados, Con demencia en estado avanzado, Con Insuficiencia Renal Crónica Terminal, Con enfermedades oncológicas en estado terminal	Visión integral e individualizada de los pacientes, Intervenir tanto sobre el paciente como sobre la familia, Debe ser atendido por diferentes especialistas según la necesidad, Se necesita mucho más que deseos de hacer el bien	Varón de 54 años con cáncer de pulmón en fase avanzada, sin expectativas de curación, Niño de 4 años con enfermedad de generativa en fase avanzada sin tratamiento curativo conocido	cuidados médicos ofrecidos cuando una enfermedad grave está en un estado muy avanzado, no tiene cura y la persona está sufriendo. Estos ayudaran a aliviar el sufrimiento para brindar una "mejor calidad de vida" del paciente.	-	-

Repetición	Fecha	Código	Seleccione los aspectos que caracterizan los Cuidados Paliativos:	Seleccione las respuestas que le parezcan correctas: "Los cuidados paliativos incluyen a los pacientes..."	Seleccione las respuestas que le parezcan verdaderas: "En los cuidados paliativos se debe tener en cuenta..."	De las siguientes situaciones seleccione aquellas que sean susceptibles a recibir cuidados paliativos:	Defina en una frase qué son los cuidados paliativos:<br>o<br>Califique la necesidad de formación de futuros profesionales no sanitarios en el área de los cuidados paliativos:	¿Cuál ha sido su experiencia en esta asignatura? (M3)	¿Cuál es el mensaje que se lleva de los cuidados paliativos?
En Momento 1 y Momento 2	08/03/2022	Turín222	Dirigidos a disminuir el sufrimiento y elevar la calidad de vida, Ofrecer apoyo a los pacientes a llevar una vida lo más activa	Con enfermedades no oncológicas crónicas en estadios avanzados, Con demencia en estado avanzado, Con Insuficiencia Renal Crónica Terminal, Con Enfermedad Pulmonar Obstructiva Crónica (EPOC) o con otra insuficiencia respiratoria, Con enfermedades oncológicas en estado terminal, Con pronóstico de vida limitado (inferior a 6 meses)	Visión integral e individualizada de los pacientes, Intervenir tanto sobre el paciente como sobre la familia	Varón de 54 años con cáncer de pulmón en fase avanzada, sin expectativas de curación, Mujer de 89 años con Insuficiencia Cardiaca avanzada que no mejora pese a tener el tratamiento adecuado, Niño de 4 años con enfermedad de generativa en fase avanzada sin tratamiento curativo conocido	Cuidados llevados a cabo en los últimos días de una persona con alguna enfermedad avanzada para aliviar su dolor	-	-
	07/02/2022	Santander812	Dirigidos a disminuir el sufrimiento y elevar la calidad de vida, Ofrecer apoyo a los pacientes a llevar una vida lo más activa, Los aspectos no espirituales no son parte de los cuidados paliativos	Con Insuficiencia Renal Crónica Terminal, Con enfermedades oncológicas en estado terminal, Con pronóstico de vida limitado (inferior a 6 meses)	Visión integral e individualizada de los pacientes, Debe ser atendido por diferentes especialistas según la necesidad, Se necesita mucho más que deseos de hacer el bien, Intervención en todos los niveles de atención asistenciales	Ninguno de los anteriores es susceptible de recibir cuidados paliativos	Es un tratamiento que se lleva a cabo en circunstancias muy concretas y extremas para proporcionar al paciente un estado de bienestar y tranquilidad, evitando el dolor causado sin posible cura.	-	-
	08/03/2022	Santander812	Dirigidos a disminuir el sufrimiento y elevar la calidad de vida, Ofrecer apoyo a los pacientes a llevar una vida lo más activa, La familia debe participar siempre en los cuidados paliativos	Con demencia en estado avanzado, Con Insuficiencia Renal Crónica Terminal, Con enfermedades oncológicas en estado terminal, Con pronóstico de vida limitado (inferior a 6 meses)	Visión integral e individualizada de los pacientes, Intervenir tanto sobre el paciente como sobre la familia, Debe ser atendido por diferentes especialistas según la necesidad, Se necesita mucho más que deseos de hacer el bien, Intervención en todos los niveles de atención asistenciales	Varón de 54 años con cáncer de pulmón en fase avanzada, sin expectativas de curación, Niño de 4 años con enfermedad de generativa en fase avanzada sin tratamiento curativo conocido	Los cuidados paliativos son un tratamiento para enfermedades crónicas o con esperanza de vida menor a 6 meses, que ayuda al paciente a que disminuya su dolor y le de mayor calidad de vida.	-	-
	19/01/2022	Pamplona123	Dirigidos a disminuir el sufrimiento y elevar la calidad de vida, Ofrecer apoyo a los pacientes a llevar una vida lo más activa, La familia debe participar siempre en los cuidados paliativos	Con enfermedades no oncológicas crónicas en estadios avanzados, Con enfermedades oncológicas en estado terminal, Con pronóstico de vida limitado (inferior a 6 meses)	Visión integral e individualizada de los pacientes, Intervenir tanto sobre el paciente como sobre la familia, Debe ser atendido por diferentes especialistas según la necesidad, Se necesita mucho más que deseos de hacer el bien, Intervención en todos los niveles de atención asistenciales	Ninguno de los anteriores es susceptible de recibir cuidados paliativos	Un medio que se emplea para poder mejorar la calidad de vida del enfermo y evitar el sufrimiento	-	-
	25/03/2022	Pamplona 123	Dirigidos a disminuir el sufrimiento y elevar la calidad de vida, La familia debe participar siempre en los cuidados paliativos	Con enfermedades oncológicas en estado terminal, Con pronóstico de vida limitado (inferior a 6 meses)	Visión integral e individualizada de los pacientes, Debe ser atendido por diferentes especialistas según la necesidad, Solo importa la voluntad del paciente y no de los cuidadores	Varón de 54 años con cáncer de pulmón en fase avanzada, sin expectativas de curación, Mujer de 89 años con Insuficiencia Cardiaca avanzada que no mejora pese a tener el tratamiento adecuado, Niño de 4 años con enfermedad de generativa en fase avanzada sin tratamiento curativo conocido	Cuidar al paciente de la mejor forma posible	-	-
En Momento 2 y Momento 3	08/03/2022	Aguascalientes123	Dirigidos a disminuir el sufrimiento y elevar la calidad de vida, Ofrecer apoyo a los pacientes a llevar una vida lo más activa, La familia debe participar siempre en los cuidados paliativos	Con enfermedades no oncológicas crónicas en estadios avanzados, Con demencia en estado avanzado, Con VIH/SIDA, Con Insuficiencia Renal Crónica Terminal, Con Enfermedad Pulmonar Obstructiva Crónica (EPOC) o con otra insuficiencia respiratoria, Con enfermedades oncológicas en estado terminal, Con pronóstico de vida limitado (inferior a 6 meses)	Visión integral e individualizada de los pacientes, Intervenir tanto sobre el paciente como sobre la familia, Debe ser atendido por diferentes especialistas según la necesidad, Se necesita mucho más que deseos de hacer el bien, Intervención en todos los niveles de atención asistenciales	Varón de 54 años con cáncer de pulmón en fase avanzada, sin expectativas de curación, Mujer de 89 años con Insuficiencia Cardiaca avanzada que no mejora pese a tener el tratamiento adecuado, Niño de 4 años con enfermedad de generativa en fase avanzada sin tratamiento curativo conocido	Disminuir el dolor o molestar del paciente, dejar que la enfermedad sea el motivo de la muerte, diferente a la eutanacia	-	-
	28/04/2022	Aguascalientes123	Dirigidos a disminuir el sufrimiento y elevar la calidad de vida, Ofrecer apoyo a los pacientes a llevar una vida lo más activa, Se ofrecen para retardar la muerte de los pacientes terminales	Con enfermedades no oncológicas crónicas en estadios avanzados, Con demencia en estado avanzado, Con VIH/SIDA, Con Insuficiencia Renal Crónica Terminal, Con enfermedades oncológicas en estado terminal, Con pronóstico de vida limitado (inferior a 6 meses)	Visión integral e individualizada de los pacientes, Intervenir tanto sobre el paciente como sobre la familia, Debe ser atendido por diferentes especialistas según la necesidad, Se necesita mucho más que deseos de hacer el bien, Intervención en todos los niveles de atención asistenciales	Varón de 54 años con cáncer de pulmón en fase avanzada, sin expectativas de curación, Mujer de 89 años con Insuficiencia Cardiaca avanzada que no mejora pese a tener el tratamiento adecuado, Niño de 4 años con enfermedad de generativa en fase avanzada sin tratamiento curativo conocido	Muy necesaria	Me encantó! Una muy bonita experiencia llena de aprendizajes	Son necesarios y me parecen muy humanos
	25/03/2022	Lisboa 555	Dirigidos a disminuir el sufrimiento y elevar la calidad de vida, La familia debe participar siempre en los cuidados paliativos	Con enfermedades no oncológicas crónicas en estadios avanzados, Con demencia en estado avanzado, Con VIH/SIDA, Con Insuficiencia Renal Crónica Terminal, Con Enfermedad Pulmonar Obstructiva Crónica (EPOC) o con otra insuficiencia respiratoria, Con enfermedades oncológicas en estado terminal, Con pronóstico de vida limitado (inferior a 6 meses)	Visión integral e individualizada de los pacientes, Intervenir tanto sobre el paciente como sobre la familia, Debe ser atendido por diferentes especialistas según la necesidad, Carácter interdisciplinario	Varón de 54 años con cáncer de pulmón en fase avanzada, sin expectativas de curación, Mujer de 89 años con Insuficiencia Cardiaca avanzada que no mejora pese a tener el tratamiento adecuado, Niño de 4 años con enfermedad de generativa en fase avanzada sin tratamiento curativo conocido	Aliviar	-	-
	28/04/2022	Lisboa555	Dirigidos a disminuir el sufrimiento y elevar la calidad de vida, La familia debe participar siempre en los cuidados paliativos	Con enfermedades no oncológicas crónicas en estadios avanzados, Con demencia en estado avanzado, Con VIH/SIDA, Con Insuficiencia Renal Crónica Terminal, Con Enfermedad Pulmonar Obstructiva Crónica (EPOC) o con otra insuficiencia respiratoria, Con enfermedades oncológicas en estado terminal, Con pronóstico de vida limitado (inferior a 6 meses)	Visión integral e individualizada de los pacientes, Intervenir tanto sobre el paciente como sobre la familia, Debe ser atendido por diferentes especialistas según la necesidad, Carácter interdisciplinario, Se necesita mucho más que deseos de hacer el bien, Intervención en todos los niveles de atención asistenciales	Varón de 54 años con cáncer de pulmón en fase avanzada, sin expectativas de curación, Mujer de 89 años con Insuficiencia Cardiaca avanzada que no mejora pese a tener el tratamiento adecuado, Niño de 4 años con enfermedad de generativa en fase avanzada sin tratamiento curativo conocido	Muy necesaria	Ha sido una experiencia muy enriquecedora y que me ha hecho abrir los ojos a una parte que siempre había evitado	Que son imprescindibles y fundamentales a la hora de lidiar con enfermedades graves, y que ayudan a los pacientes a tener un final de vida digna.

Repetición	Fecha	Código	Seleccione los aspectos que caracterizan los Cuidados Paliativos:	Seleccione las respuestas que le parezcan correctas: "Los cuidados paliativos incluyen a los pacientes..."	Seleccione las respuestas que le parezcan verdaderas: "En los cuidados paliativos se debe tener en cuenta..."	De las siguientes situaciones seleccione aquellas que sean susceptibles a recibir cuidados paliativos:	Defina en una frase qué son los cuidados paliativos:<br>o<br>Califique la necesidad de formación de futuros profesionales no sanitarios en el área de los cuidados paliativos:	¿Cuál ha sido su experiencia en esta asignatura? (M3)	¿Cuál es el mensaje que se lleva de los cuidados paliativos?
	25/03/2022	SanSebastian123	Dirigidos a disminuir el sufrimiento y elevar la calidad de vida, La familia debe participar siempre en los cuidados paliativos	Con enfermedades no oncológicas crónicas en estadios avanzados, Con demencia en estado avanzado, Con VIH/SIDA, Con Insuficiencia Renal Crónica Terminal, Con Enfermedad Pulmonar Obstructiva Crónica (EPOC) o con otra insuficiencia respiratoria, Con enfermedades oncológicas en estado terminal, Con pronóstico de vida limitado (inferior a 6 meses)	Visión integral e individualizada de los pacientes, Intervenir tanto sobre el paciente como sobre la familia, Debe ser atendido por diferentes especialistas según la necesidad, Carácter interdisciplinario, Intervención en todos los niveles de atención asistenciales	Varón de 54 años con cáncer de pulmón en fase avanzada, sin expectativas de curación, Mujer de 89 años con Insuficiencia Cardiaca avanzada que no mejora pese a tener el tratamiento adecuado, Niño de 4 años con enfermedad de generativa en fase avanzada sin tratamiento curativo conocido	Alivio del sufrimiento y mejorar la calidad de vida del paciente	-	-
	28/04/2022	SanSebastian123	Dirigidos a disminuir el sufrimiento y elevar la calidad de vida, Los aspectos no espirituales no son parte de los cuidados paliativos	Con enfermedades no oncológicas crónicas en estadios avanzados, Con demencia en estado avanzado, Con VIH/SIDA, Con Insuficiencia Renal Crónica Terminal, Con Enfermedad Pulmonar Obstructiva Crónica (EPOC) o con otra insuficiencia respiratoria, Con enfermedades oncológicas en estado terminal, Con pronóstico de vida limitado (inferior a 6 meses)	Visión integral e individualizada de los pacientes, Intervenir tanto sobre el paciente como sobre la familia, Debe ser atendido por diferentes especialistas según la necesidad, Carácter interdisciplinario, Se necesita mucho más que deseos de hacer el bien, Intervención en todos los niveles de atención asistenciales	Varón de 54 años con cáncer de pulmón en fase avanzada, sin expectativas de curación, Mujer de 89 años con Insuficiencia Cardiaca avanzada que no mejora pese a tener el tratamiento adecuado, Niño de 4 años con enfermedad de generativa en fase avanzada sin tratamiento curativo conocido	Muy necesaria	Para mí ha sido muy gratificante cursar esta asignatura ya que considero que he aprendido acerca de aspectos que puedo aplicar en mi día a día	Son unos cuidados imprescindibles y fundamentales a los cuales todas las personas deben de tener acceso
