# Supplementary material for: Educational innovation as a communication strategy in palliative care: A study protocol and preliminary results
Source: PLoS One. 2023 Jun 9;18(6):e0286343. doi: 10.1371/journal.pone.0286343 (PMC10256175; doi:10.1371/journal.pone.0286343)
Supplement: S2 File — (PDF) [file pone.0286343.s005.pdf]

Módulo IV

El discurso social de cuidar

Antes de empezar, asegúrate de haber **leído y firmado** la hoja de información. A continuación tendrás que **responder 5 preguntas** relacionadas con los discursos que escucharás en clase y sobre tu conocimiento sobre los cuidados paliativos.

Discursos

¿Cuál de estos discursos **te ha informado más**? Marca con una X tu respuesta.

1 | 2 (X) | 3 | 4 | 5

¿Cuál de los discursos **te ha llamado más a la acción**? Marca con una X tu respuesta.

1 | 2 | 3 | 4 (X) | 5

Conocimiento

Define, con tus palabras, tras lo que ha escuchado, **qué son los cuidados paliativos**:

Los cuidados paliativos son unos tratamientos médicos que están destinados a pacientes con una enfermedad terminal con el objetivo de reducir, al máximo posible, su sufrimiento.

¿**Cambiarías algo del discurso** que escribiste en COE2 una vez escuchado esto? Marca con una X tu respuesta.

Sí (X) | No

Si es que sí, ¿**qué cambiarías**? Por favor, explícalo de la forma más completa posible.

Seguramente, haría como muchos de los que han hecho los discursos e intentaría contactar con una persona terminal - de manera personal - para potenciar mi "ethos".

Módulo IV

El discurso social de cuidar

Antes de empezar, asegúrate de haber **leído y firmado** la hoja de información. A continuación tendrás que **responder 5 preguntas** relacionadas con los discursos que escucharás en clase y sobre tu conocimiento sobre los cuidados paliativos.

Discursos

¿Cuál de estos discursos **te ha informado más**? Marca con una X tu respuesta.

1 — 2 [X] — 3 — 4 — 5

¿Cuál de los discursos **te ha llamado más a la acción**? Marca con una X tu respuesta.

1 — 2 — 3 [X] — 4 — 5

Conocimiento

Define, con tus palabras, tras lo que ha escuchado, **qué son los cuidados paliativos**:

Son cuidados especiales que se enfocan a personas que no tienen la posibilidad de curarse de su enfermedad. Tratan de apoyo, tanto enfocado a la enfermedad, como a consecuencias psicológicas; tanto al paciente como a las familias

¿**Cambiarías algo del discurso** que escribiste en COE2 una vez escuchado esto? Marca con una X tu respuesta.

Sí [X] — No

Si es que sí, ¿**qué cambiarías**? Por favor, explícalo de la forma más completa posible.

Ahora, sí, porque una vez se recibe conocimiento acerca de los cuidados paliativos, es más fácil ponerse a escribir. Este tema es complejo de hablar y de comunicar, porque es sobre algo muy emotivo, por lo que hay que hilar muy fino. Hablaría más de lo que es un cuidado paliativo y métodos para ayudar y actuar antes de encontrarse ante la enfermedad.
