# Supplementary material for: Educational innovation as a communication strategy in palliative care: A study protocol and preliminary results
Source: PLoS One. 2023 Jun 9;18(6):e0286343. doi: 10.1371/journal.pone.0286343 (PMC10256175; doi:10.1371/journal.pone.0286343)
Supplement: S3 File — (PDF) [file pone.0286343.s006.pdf]

Focus group - 2 de junio

Guion

Introductorias:

¿Qué fue lo que más les gustó de impartir o recibir esta asignatura?

De exploración:

¿Impartir o recibir esta asignatura te produjo algún sentimiento?

- ¿Qué cosa en concreto de la asignatura te provocó ese sentimiento?

Si pudieras cambiar 3 cosas de la asignatura, ¿qué cosas cambiarías?

- ¿Crees que tus compañeros/otros profesores tendrían esa misma valoración?

De salida (discusión final):

¿Por qué recomendarían esta asignatura?

¿Hay algún tema que les gustaría tocar y no lo hicimos?

Notas de campo investigador 1

¿Qué fue lo que más les gustó de impartir o recibir esta asignatura?

P1:
- Le gustó que se hablara de emociones de forma natural
- Que hubiera poca gente para hablar mejor

P2:
- Poca gente, podíamos hablar más
- Le gustó la clase de CC
- Le gustó el stay room porque ves a la persona como persona
- Le gustó ir a la Cruz Roja porque abrió puertas a ver cuidado de otra manera

P3:
- Le gustó ver algo no relacionado con su carrera
- Le gustó ver cómo cuidar a los demas
- Le gustaron los testimonios

P4:
- Grupos pequeños hacia que fuera un dialogo fácil
- Grupos pequeños permitían amoldar a clases o a necesidades y a solicitudes de alumnos
- Aprendió también con sus alumnos

P5:
- Experiencia difícil por darle clase a gente de otro entorno
- Fue un reto dirigirse a personas que no tienen que ver con salud
- Le hubiera gustado ver cómo era el resto del curso
- Le gusto ayudar a normalizar las situación expuestas en el curso

P6:
- Le gustó que fueron alumnos de distintas carreras
- Le gustó poder leer "sus pensamientos"

P5:
- Le gustó ver sus reflexiones que no eran distintas a las de los médicos

¿Impartir o recibir esta asignatura te produjo algún sentimiento?

P2:
- Le produjo sentimientos tanto tanto positivos como negativos
- Había videos fuertes que producían emociones complicadas
- Fue difícil, y es importante modular. Una amiga suya ya no quería regresar por cuestiones personales.
- Pero lo demás de la asignatura fue positivo
- Le gustó ver a personas que salían de situaciones difíciles
- Sintió esperanza al ver que se puede tener calidad de vida

P1:
- Salía con "chispita" de la clase, como motivado
- Le provocaba llamar a su abuela para acompañar su vejez
- Había muchos sentimientos en las sesiones en las que se hablaba de la muerte
- Se había olvidado de su tío con esclerosis y esto lo acercó

P3:
- A veces le damos mucha atención a sentimientos
- Tener cuidado con generalizar los sentires
- No porque llore más o menos la realidad es distinta
- Hay que saber gestionar sentimientos

P4:
- Videos que eran fuertes generaban sentimientos en alumnos
- Ella vivió el Alzheimer y se le hizo complicado dar la clase
- Le ha emocionado ver que los alumnos tengan una realidad similar a la suya

P5:
- Le gustó lo que dijo DF de querer acompañar a abuela después de clase, para ella el objetivo estaba conseguido
- Si alguien se emociona un poco más al ver uno de los videos o testimonios está bien
- Situaciones complicadas cuestan pero ayuda sentir, no es malo

  DF (como respuesta a AL)
  - Videos buena manera de comunicar y dialogar

P6:
- Sintió vertigo: es muy difícil cómo contar esto, cómo lo hacemos para que la gente lo haga bien
- Sensación general de que compartimos algo en el aula

Si pudieras cambiar 3 cosas de la asignatura, ¿qué cosas cambiarías?

P3:

- Le hubiera gustado que hubiera casos prácticos de poner en situación y explicar
- Echó en falta más objetividad, ir a la razón, más bibliografía, explicar cosas como eutanasia

P4 (Respuesta a P3)

- Sí hay bibliografía, pero queríamos llegar a conclusiones desde experiencia

P2 (Respuesta a P3)

- Lo que facilitó aprender era justo que no fuera una lista de datos
- Si sería util tener más bibliografia pero peligroso solo hablar solo de cosas concretas

P2:

- Brindaría herramientas psicológicas para gestionar sentimientos
- Daría más recursos para mejorar cuidado, pero que no sea lo central de la asignatura
- La asignatura te hace reflexionar sobre la vida, le gustó esa experiencia

DF (respuesta a IM)

- Está de acuerdo con lo de dar más recursos para mejorar cuidado

P1:

- Le faltó una parte más creativa de la asignatura
- Difícil poner todo lo que aprendió en un discurso, pero entiende por qué es importante
- Le hubiera gustado más platicarlo una dinámica de platicarlo (como focus group)

P2 (Respuesta a P1):

- Para ella sí fue muy util la clase de comunicación
- Valora la corrección
- Igual le gustaría más horas de clase de comunicación
- Pero sí le costó bastante

P3 (respuesta a P1 y P2):

- Importante saber hablar y saber escribir

P6:

- Más horas para dar su clase (comunicación)

- Cambiaría el aula por una más grande
- Cambiaría la ortografía de los alumnos

P5:

- Hay problema de puntualidad para comenzar la clase
- Le gustó la idea de poner ejemplo (caso práctico) y hablarlos en clase

  P6 (respuesta a P5):

  - Sugiere cambiar a horario de tarde

  P1 (respuesta a P6):

  - Le gusta el horario actual porque acababa el día feliz y clase le daba energía, y más tarde estarían más cansados

¿Por qué recomendarías Cuidado y Sociedad?

P1:

- Te sensibiliza
- Es útil para tu vida
- No tiene examen

P2:

- Que no haya examen quita peso de encima, estás más receptiva
- Te enseña cosas que te sirven
- Es ligera, porque no vas a memorizar
- Modalidades diferentes, perspectivas diferentes, actividades diferentes

P3:

- Para que los que las cursan se pongan a pensar sobre el tema
- Es abierta y receptiva

Notas de campo investigador 2

Lo que más gustó
- Hablar entre alumnos
- Ver como se sorprendían a los alumnos
- Grupos pequeños
- Libertad para hablar de la muerte
- Hacer reflexionar
- El stay-room (oportunidad distinta de aprender sobre el cuidado con una historia real)
- Estar en contacto con la cruz roja
- Hacer voluntariado
- Los testimonios
- Distintos grados

Sentimientos
- Muchos sentimientos (positivos y negativos)
- Motivación
- Esperanza
- "Llamar a mi abuela" (acompañar)
- "Necesidad de contactar a mi tío que tenía una enfermedad"
- "Esto es algo muy difícil"
- "Realidad es dura"

- Ahora me llevo más de cómo quiero vivir mi vida
- Mejor practicar el ensayo que escribirlo
    - (respuesta) La parte escrita fue importante

Por qué te metiste:
- Muchas cosas distintas
- Porque era solo atender y reflexionar (no examen)
- Vas a escuchar y aprender
- Te sensibiliza / Te cambia
- Te sirve para la vida
- Solo tienes que dedicar el tiempo de la asignatura
- La intension de la asignatura es genuina

- Está abierta a sugerencias
- Tenía otras expectativas
- Pararte para esucuchar sobre este tema

Profesores:

- Adaptar módulo 4 a su punto de vista
- Un video
- Uso de metaforas
- Enseñar CP a estudiantes de no medicina es util

Qué harían con asignatura

- Mantener grupos pequeños
- Tener alumnos de distintos grados
- Llevarles a la acción
- Una asignatura real y compleja
- Hay que darles recursos para lidiar con eso
- Muchas cosas para poco tiempo
- Módulo 4 provoca discusión
- Las reflexiones de los estudiantes no son tan distintas que las de medicina
- Videos muy impactantes
- Justificar mejor los contenidos (no caer en tantos sentimientos)
- Pondrían casos prácticos
- Técnicas y herramientas para manejar sentimientos
- Más horas
