# Supplementary material for: Educational innovation as a communication strategy in palliative care: A study protocol and preliminary results
Source: PLoS One. 2023 Jun 9;18(6):e0286343. doi: 10.1371/journal.pone.0286343 (PMC10256175; doi:10.1371/journal.pone.0286343)
Supplement: Attachment — Submitted filename: Response to reviewers.pdf [file pone.0286343.s008.pdf]

March 3, 2023

Dear Reviewers and Editors of PLOS ONE:

Allow me to start by extending my sincerest gratitude to the editors who have invested time and effort in finding reviewers for this paper, as well as to the reviewers who have agreed to take upon such request. It carries a lot of weight for us to know that the research we do ignites interest beyond the communication field. Thus, it is with great pleasure that we resubmit our article for further consideration.

I would also like to thank all of you beforehand for all the feedback and suggestions. You have raised important questions and I will proceed to enumerate the answers in the following letter.

Reviewer #1:

Dear researcher,
Your effort and motivation in doing this research is commendable. Please pay attention to the following points and correct them.

1- In part 3.3, why did you consider the evaluation time 5 months after the first stage? Is there a specific reference or reason?

We have supplemented the section 3.4.4 with explanations as to why a 5-month time frame was selected (page 17, paragraph 2).

The 5-month timeframe was selected taking into consideration that the course grades will be made public in early June. Hence, we will be recontacting students no sooner than in November of 2022. Thus, by the time we recontact the students, some of them will be finishing their second-to-last semester in university. This is significant for two reasons. First and foremost, the recontact form they received in the Moment 1 of evaluation allowed communication via their university email. If we wait longer, some of the emails may become inoperative. Secondly, sending invitations with a semester's leeway allows us to have plenty of time to schedule

interviews knowing that the students are still in the same geographic location, facilitating face-to-face interviews or at least with similar schedules to ours.

2- In the first stage of the evaluation, what ways did you use to reduce the error in selecting the participants? For example, did you examine participants' previous knowledge or exposure to palliative care? How? Did you exclude such people from the research? If so, please mention it.

We have redrafted and added further information on the section 3.4.1 (page 14, paragraph 2) to establish a clearer focus.

The initial PCKQ is meant to examine participants previous knowledge on palliative care. If any student shows a formidable knowledge on the topic, they will not be excluded from the study. Their performance will simply be recorded, and any improvement will be taken into consideration.

3- In part 4.2, or the focus group, did you assess the previous knowledge of 2 guests about palliative care? Because at the end of the article, you have evaluated their learning about palliative care and you have stated one of the most important results of your research on the basis that: "Our participants' knowledge about palliative care has increased and their skills in transferring This knowledge to others (guests), has been acceptable.

Regarding the section 4.2 on the Message Transmission Experiment, we have elaborated on the assessment of the previous knowledge (page 23, paragraph 2) and we also acknowledge in section 7 that a better assessment of previous knowledge of recipients needs to be done in future interventions (page 30, paragraph 2).

1) During the last class of the course, five students were selected to read out loud their speech in front of their classmates and two guests. The guests were two first-year students not enrolled in the course, who have previously display little or no knowledge regarding palliative care in an assignment delivered for another university course. After hearing all the speeches, they were asked to define palliative care.

2) After completing the course, some of the students appeared to be able to explain what palliative care is to guest students with little or no experience in the topic. Nonetheless, in future evaluations more work needs to

be done in assessing the efficiency of the message transmission. A more accurate assessment of the recipients' previous knowledge on the topic needs to be carried out.

Regarding the section 4.3 on the Focus Group, we have not done any previous knowledge assessment as our sense is that this activity did not aim to evaluate knowledge of any sort. We have added a clarification of our scope and give further information in section 3.4.3 (page 13, paragraph 2).

The purpose of this focus group is not to evaluate the knowledge of the participants, but to discuss the strengths and weaknesses of the methodology, the content and other aspects of the course.

5- In section 4.3, although in the abstract of the article, you stated the method of data analysis of focus groups, it would be better if you mentioned this first in this part of the article.

We agree with your assessment, and we have reflected this comment by adding the information in section 3.4.3 (page 16, paragraph 3).

The focus groups will later be transcribed, and thematic content analysis of the material will be carried out.

We have also incorporated your comments regarding the results by redrafting parts of the section 4.1 Palliative Care Knowledge Questionnaire (page 20) and section 7 Conclusions (page 30).

Reviewer #2:

-Methods: determine of date and place of study.

We agree with you and have incorporated this suggestion in section 3 (page 11, paragraph 2).

The project started on November 2021 and will continue until September 2024 in Spain. The pilot study took place on the second semester of the academic course 2021-2022, starting on January 2022 (Fig 2). However, enrollment to the course took place five months before, in August of 2022, when the university course registration period was open.

-Findings are ambiguous and not explained.

We have elaborated on various points throughout section 4. Preliminary results (page 20). We hope these revisions provide more clarity.

Introduction

- In the introduction, the importance of the study and the existing gap should be explained

We have revised the text (page 5, paragraph 2) to reflect the existing gap and the importance of the study.

Furthermore, palliative care has a communication problem, as 54 years after its creation the concept is still misunderstood. Palliative care is currently perceived as "death" or "end of life”, and that misconception continues to generate feelings of bereavement, fear, and anxiety in society [7,8]. In Spain, the media may play an important role in this misunderstanding. An analysis of 600 Spanish newspapers (2009-2014) concluded that information on palliative care does not reflect its true nature, as the narratives used to talk about it are based on ideological and moral content such as the debate of issues like euthanasia [9]. The importance of this study stems from the need to find alternative routes to reach an alienated audience. Society receives a distorted version of the palliative care message and identifying a more direct channel of communication could be a solution.

-The discussion needs more analysis and interpretation

We agree and we have elaborated on several points throughout section 5. Discussion (page 21).

-The written format of the article is not suitable in different sections. For example, the introduction is long and the discussion is short

We have now both reduced the length of the Introduction and elaborate on the Results and Discussion. We think these changes now better the balance of the different sections. We hope that you agree.

Editor comments:

1. Please ensure that your manuscript meets PLOS ONE's style requirements, including those for file naming.

We have ensured that our manuscript meets PLOS ONE'S style requirements following the guidelines that you provided.

2. During your revisions, please note that a simple title correction is required: Study protocol requires the word ""protocol' to be included in the title. Please ensure this is updated in the manuscript file and the online submission information.

We have now added "Study protocol" to the title and ensure that it is updated both in the online platform and the manuscript.

3. We note that the grant information you provided in the 'Funding Information' and 'Financial Disclosure' sections do not match. When you resubmit, please ensure that you provide the correct grant numbers for the awards you received for your study in the 'Funding Information' section.

We have changed the information for the sections to match.

4. We note that you have indicated that data from this study are available upon request. PLOS only allows data to be available upon request if there are legal or ethical restrictions on sharing data publicly. For more information on unacceptable data access restrictions, please see… In your revised cover letter, please address the following prompts: […]

b) If there are no restrictions, please provide the minimal anonymized data set necessary to replicate your study findings as either Supporting Information files or to a stable, public repository and provide us with the relevant URLs, DOIs, or accession numbers. For a list of acceptable repositories, please see… We will update your Data Availability statement on your behalf to reflect the information you provide.

We have provided the minimal anonymized data set necessary to replicate the study findings as Supporting Information files.

5. Please ensure that you refer to Figures 1 and 2 in your text as, if accepted, production will need this reference to link the reader to the figure.

We have referred to Figures 1 and 2 in the text.

6. Please review your reference list to ensure that it is complete and correct. If you have cited papers that have been retracted, please include the rationale for doing so in the manuscript text, or remove these references and replace them with relevant current references. Any changes to the reference list should be mentioned in the rebuttal letter that accompanies your revised manuscript. If you need to cite a retracted article, indicate the article's retracted status in the References list and also include a citation and full reference for the retraction notice.

We have reviewed the reference list to ensure that it is complete and correct.

Additional Editor's comments:

Our Initial Quality Check of your submission has now taken place. As a result, we need you to address the following points before your manuscript can progress any further:
1. The trial must be registered prior to submission of your protocol in one of the publicly accessible registries approved by the WHO or ICMJE (International Committee of Medical Journal Editors).
2. The name of the registry and the trial or study registration number must be included in the Abstract.

The trial has been registered, and the registry and registration number have been included in the Abstract (page 2).

This study is registered on the ISRCTN Registry under the name "Can a university course help communicate palliative care?" (Registration number: ISRCTN10236642).

3. A copy of the protocol that was approved by the ethics committee must be submitted as a supplementary information file. Please provide an additional English translation if the original document is not in English. Please note that the protocol will be published with the manuscript if accepted.

A copy of the protocol that was approved by the ethics committee and its translation have been included as supplementary information files (page 30).

4. A SPIRIT schedule of enrollment, interventions, and assessments must be included as the manuscript's Figure 1, and a completed SPIRIT checklist must be uploaded as Supporting Information file S1.

A SPIRIT schedule of enrollment, interventions, and assessments was included as Figure 2 (page 11), as it was our sense that the figure was a better fit for section 3. Method.

The project started on November 2021 and will continue until September 2024 in Spain. The pilot study took place on the second semester of the academic course 2021-2022, starting on January 2022 (Fig 2). However, enrollment to the course took place five months before, in August of 2022, when the university course registration period was open.

We would like to thank you one more time for allowing us to enhance our manuscript with your comments and queries. We have diligently incorporated your feedback and hope that these revisions persuade you to accept our submission.

Sincerely,

Ana Paula Salas
Corresponding Author
PhD Candidate
ATLANTES Global Observatory of Palliative Care
University of Navarra
asalasmo@unav.es
