# Supplementary material for: Educational innovation as a communication strategy in palliative care: A study protocol and preliminary results
Source: PLoS One. 2023 Jun 9;18(6):e0286343. doi: 10.1371/journal.pone.0286343 (PMC10256175; doi:10.1371/journal.pone.0286343)
Supplement: Attachment — Submitted filename: comment.docx [file pone.0286343.s007.docx]

Abstract

- Methods: determine of date and place of study.

-Findings are ambiguous and not explained.

Introduction

- In the introduction, the importance of the study and the existing gap should be explained

The discussion needs more analysis and interpretation

The written format of the article is not suitable in different sections. For example, the introduction is long and the discussion is short
