# Supplementary material for: Educational innovation as a communication strategy in palliative care: A study protocol and preliminary results
Source: PLoS One. 2023 Jun 9;18(6):e0286343. doi: 10.1371/journal.pone.0286343 (PMC10256175; doi:10.1371/journal.pone.0286343)
Supplement: Attachment — Submitted filename: reviewer.docx [file pone.0286343.s010.docx]

Dear Editor,

The article has been revised and changes based on comments are accepted.
